# Necessity of integrated genomic analysis to establish a designed knock-in mouse from CRISPR-Cas9-induced mutants

**DOI:** 10.1038/s41598-022-24810-5

**Published:** 2022-11-27

**Authors:** Masahide Yoshida, Tomoko Saito, Yuki Takayanagi, Yoshikazu Totsuka, Tatsushi Onaka

**Affiliations:** 1 Division of Brain and Neurophysiology, Department of Physiology, Jichi Medical University, 3311-1 Yakushiji, Shimotsuke, Tochigi 329-0498 Japan; 2 Institute of Immunology Co., Ltd., 1198-4 Iwazo, Utsunomiya, Tochigi 321-0973 Japan

**Keywords:** Biological techniques, Biotechnology, Molecular biology

## Abstract

The CRISPR-Cas9 method for generation of knock-in mutations in rodent embryos yields many F0 generation candidates that may have the designed mutations. The first task for selection of promising F0 generations is to analyze genomic DNA which likely contains a mixture of designed and unexpected mutations. In our study, while generating Prlhr-Venus knock-in reporter mice, we found that genomic rearrangements near the targeted knock-in allele, tandem multicopies at a target allele locus, and mosaic genotypes for two different knock-in alleles occurred in addition to the designed knock-in mutation in the F0 generation. Conventional PCR and genomic sequencing were not able to detect mosaicism nor discriminate between the designed one-copy knock-in mutant and a multicopy-inserted mutant. However, by using a combination of Southern blotting and the next-generation sequencing-based RAISING method, these mutants were successfully detected in the F0 generation. In the F1 and F2 generations, droplet digital PCR assisted in establishing the strain, although a multicopy was falsely detected as one copy by analysis of the F0 generation. Thus, the combination of these methods allowed us to select promising F0 generations and facilitated establishment of the designed strain. We emphasize that focusing only on positive evidence of knock-in can lead to erroneous selection of undesirable strains.

## Introduction

Gene targeting, which transduces a mutation in a specific endogenous gene, has been broadly used to generate animal models for understanding physiological or pathological mechanisms. Homologous recombination in embryonic stem (ES) cells has been classically used to obtain gene-targeted rodents^1^. However, with this method, it takes nearly one year to obtain a genetically targeted mouse, including the time required to produce correctly targeted ES cell clones and acquiring animals capable of reproduction from chimeric mice. In contrast, genome editing methods such as zinc finger nuclease (ZFN)^2^, transcription activator-like effector nucleases (TALEN)^3^, and CRISPR-Cas9 ribonucleoprotein complexes^4,5^, are promising approaches for obtaining gene-targeted rodents in a limited period of time and for accelerating biological and medical research. Development of the CRISPR-Cas9 method is particularly remarkable because of its simplicity and efficiency^6^. During homologous recombination in ES cells, a gene targeting vector is electroporated into ES cells and drug-resistant ES cells are screened. Southern blotting or PCR analysis is then used to further select ES cell clones that have been recombined correctly. Lastly, the ES cell clones are microinjected into blastocysts to obtain germline-transmitted chimeric rodents^7^. In contrast, in the CRISPR-Cas9 method the combination of CRISPR RNA (crRNA), including the recognition sequence complementary to the targeting genomic sequence, and trans-activating CRISPR RNA (tracrRNA), or single guide RNA (sgRNA), and Cas9 nuclease are co-injected into fertilized eggs and the target gene is directly modified within embryos^8,9^. While it only takes three months to obtain adult candidate rodents, genetic variation must be investigated using genomic DNA extracted from somatic tissues of individual rodents.

In the past few years, targeted knock-in methods have been developed which involve administering donor DNA template along with a component of the CRISPR-Cas9 complex. The CRISPR-Cas9 complex recognizes a specific sequence and cleaves the double-stranded DNA (dsDNA) adjusted to the PAM sequence. In mammalian cells, site-specific dsDNA breaks are repaired by nonhomologous end joining (NHEJ), homology-directed repair (HDR), or microhomology-mediated end joining (MMEJ) mechanisms^10,11^. Knock-in events can occur when repair of dsDNA breaks is performed using donor DNA template with two homology arms on either side of the transgene of interest^12^. Many researchers in the genome editing community have been endeavoring to improve the efficiency and specificity of CRISPR-Cas9-based knock-in methods in rodent embryos^13–21^. However, some obstacles remain in producing an accurate and rapid knock-in rodent model using these techniques. It has recently been reported that multiple tandem integrations at a target locus are frequently observed in F0 generation mice derived from zygotes injected with a combination of two guide RNAs and one donor DNA containing two loxP sequences for generation of conditional knock-out mice. In addition, the mosaic genotypes of the F0 generation have been revealed by analysis of F1 generation mice^22,23^. It was also noted that conventional PCR analysis, which is frequently used for genotype confirmation, failed to identify such multiple integration events in most cases, leading to a high rate of erroneous identification of mutants as correct single copy recombination events^23^.

The knock-in method using CRISPR-Cas9 can enable the insertion of reporter genes and the generation of preclinical rodent models such as models of triplet disease and cancer by insertion of causative sequences^24–26^. It is thus critical to obtain the designed and precise knock-in rodents in order to reproduce these human pathological conditions and to elucidate the molecular mechanisms underlying diseases and physiological functions. With the CRISPR-Cas9 method, many candidate rodents that may have the designed mutation can be generated. In previous reports, targeted integration was identified in up to 67% of the F0 generation by conventional PCR analysis^17,18^. Analysis of the F1 generation, bred by mating all candidate F0 mice, results in unnecessary breeding of research animals and requires a lot of time, effort, and space, which creates a bottleneck for the CRISPR-Cas9 method. Conversely, if the number of F0 generation candidates is small, a decision must be made whether to perform additional injections to obtain more F0 generation animals. If the need for additional F0 mice could be determined without having to wait for the results from F1 generation analysis, it would save a great deal of time and effort. Whole genome sequencing seems to be the best method to validate the results of genome editing; however, it has been noted that whole genome sequencing using only the tail genome is not effective in mosaic animals^27^. It has also been noted that single-cell genome sequencing with somatic or germline cells requires further technical improvements in genome coverage, accuracy, and throughput^28,29^. In general, harvesting F0 generation germline cells requires invasive procedures that carry the risk of reduced fertility. Therefore, the first choice is to analyze genomes obtained from mosaic somatic cells. Although there are several methods for analyzing genome structure, the only way to identify rodents with the desired knock-in allele from a mixture of designed and unpredictable mutants is to combine multiple methods. However, it is not clear which combination of methods is most effective for identifying possible mutations produced by the CRISPR-Cas9-based knock-in method and for establishing a strain that has the designed mutation.

In this study, we generated prolactin-releasing peptide receptor (Prlhr)-Venus knock-in reporter mice by a CRISPR-Cas9 method with one guide RNA and one long single-stranded DNA (lssDNA) making use of HDR mechanisms. We performed five different genome structure analyses, including the recently developed Rapid Amplification of Integration Sites without Interference by Genomic DNA contamination (RAISING) method, and compared the results over three generations to establish the desired strain. These analyses revealed that genomic rearrangement in the vicinity of the targeted knock-in allele as well as multicopy and mosaic genotypes occurred in the F0 generation; however, we were able to obtain a promising F0 generation by eliminating undesired mutations to establish the designed strain.

## Results

### Generation of CRISPR-Cas9-based Prlhr-Venus knock-in mice with lssDNA and one sgRNA

**Figure 1:**
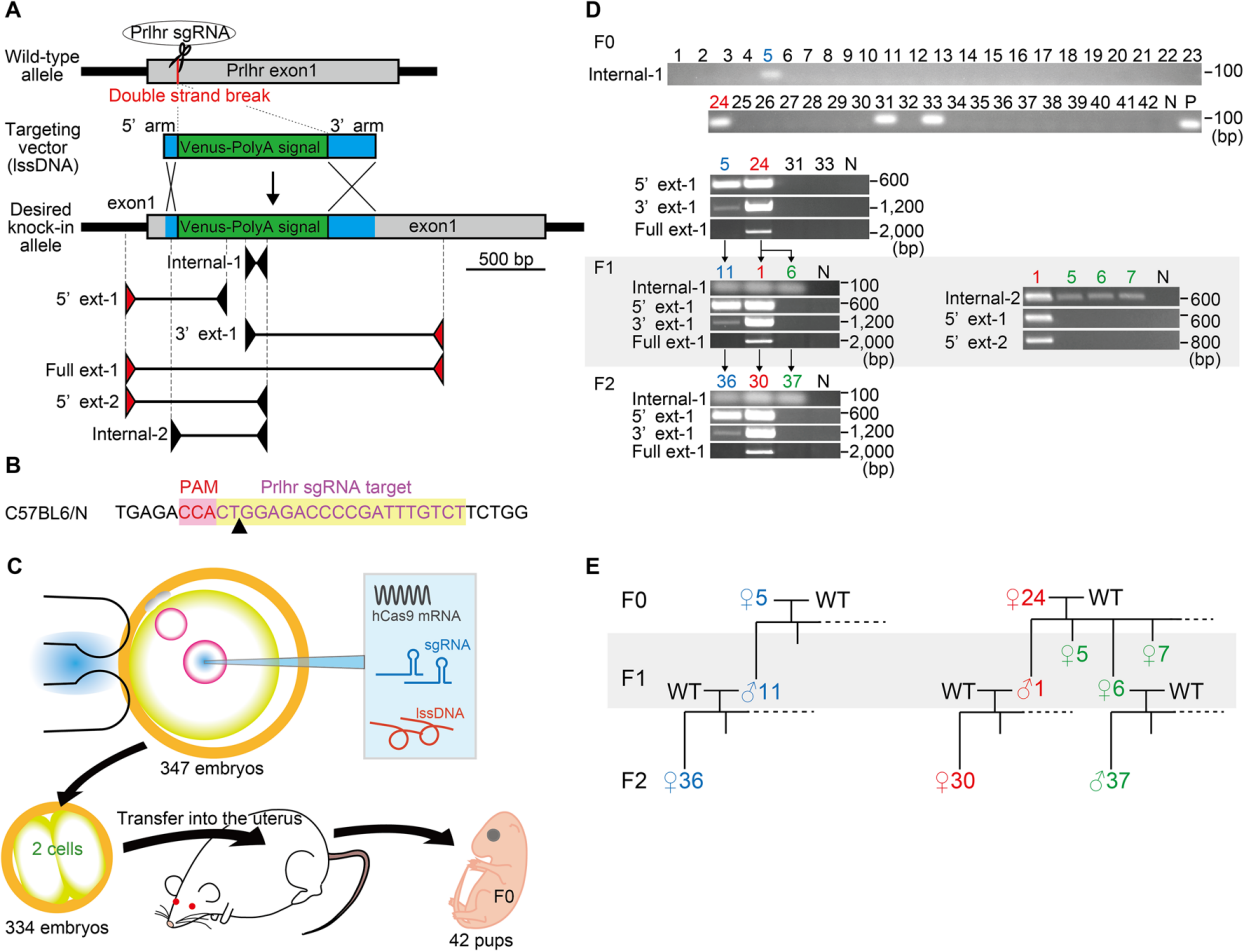
CRISPR-Cas9-mediated knock-in strategy at the prolactin releasing-peptide receptor (Prlhr) locus and analysis of Venus integration using conventional PCR. (**A**) Schematic representation of the wild-type mouse Prlhr genomic locus, sgRNA targeting site, targeting vector containing the Venus-polyadenylation signal, and primer sets (internal primer pair, Internal-1 and Internal-2; primer external to the targeting vector and internal primer pairs, 5’ext-1, 3’ext-1, 5’ext-2; primer pair external to the targeting vector, Full ext-1). Red and black arrows show the primer external to the targeting vector and internal primer, respectively. (**B**) Target sequence of Prlhr sgRNA on the C57BL6/N genome. (**C**) Schematic representation of hCas9 mRNA, sgRNA, and lssDNA co-injection into mouse embryos to generate founders (F0) with the knock-in mutation. (**D**) Conventional PCR analysis of genomic DNA from the F0, F1 and F2 generations using each primer pair. Oxytocin receptor-Venus knock-in heterozygous and wild-type mice were used for positive (P) and negative (N) controls, respectively. Black arrows show the parent–offspring relationships. Uncropped gel images are presented in Supplemental Fig. 9A. (**E**) Family trees of numbers 5 and 24 of the F0 generation.

We sought to generate Prlhr-Venus knock-in mice by inserting a Venus-SV40 polyadenylation signal cassette into the gene encoding Prlhr (Fig. 1A). The target site of sgRNA was 24 bp downstream of the Prlhr initiation codon to produce 9 amino acids of a Prlhr and Venus fusion protein (Fig. 1B). We microinjected a mixture of human codon-optimized Cas9 nuclease (hCas9) mRNA, sgRNA, and lssDNA into 347 embryos and transferred 334 two-cell embryos into pseudopregnant female mice. From this, we obtained 42 pups of the F0 generation from 11 mothers (Fig. 1C).

### Conventional PCR analysis

When using the CRISPR-cas9 method for gene targeting in embryos, the initial step in mutation confirmation is conventional PCR of the F0 generation because it is convenient and sensitive. We first performed PCR analysis with an internal primer pair within the Venus-SV40 polyadenylation signal cassette. The PCR analysis revealed that 4 of the 42 pups obtained showed positive amplification (upper panel of Fig. 1D, Internal-1). We then used primers external to the targeting vector and internal primers in combination. PCR products of the 5’ and 3’ regions representing a potentially targeted allele were detected in F0 animals 5 and 24 (second panel of Fig. 1D, 5’ ext-1 and 3’ ext-1). The PCR product generated by the primer pair external to the targeting vector was only found in animal 24 of the F0 generation (second panel of Fig. 1D, Full ext-1).

We crossed F0 numbers 5 and 24 with wild types to obtain the F1 generation (Fig. 1E). The F1 and F2 progeny of F0 number 5 showed precisely the same detection pattern as F0 number 5. However, the progeny of F0 number 24 were divided into two types. The PCR products of the 5’ and 3’ regions were detected in number 1 but not in number 6 of the F1 (left third panel of Fig. 1D, 5’ ext-1 and 3’ ext-1). We performed additional analysis of the offspring using other primer pairs. The PCR products of the 5’ regions from the external-internal primer pairs were not detected in numbers 5, 6 and 7 of the F1 (right third panel of Fig. 1D, 5’ext -1 and 2). The PCR product amplified from the primer pair external to the targeting vector was found in number 1 of the F1 (left third panel of Fig. 1D, Full ext-1). The detection pattern of PCR products in the F2 was exactly the same as that of their parents (fourth panel of Fig. 1D). Two candidate F0 mice with a Venus insertion in the Prlhr locus were detected by conventional PCR. However, analysis of their F1 and F2 generation progeny showed that F0 number 24 was a mosaic with at least two types of Venus insertions that could be separated by PCR analysis.

### Droplet digital PCR analysis

**Figure 2:**
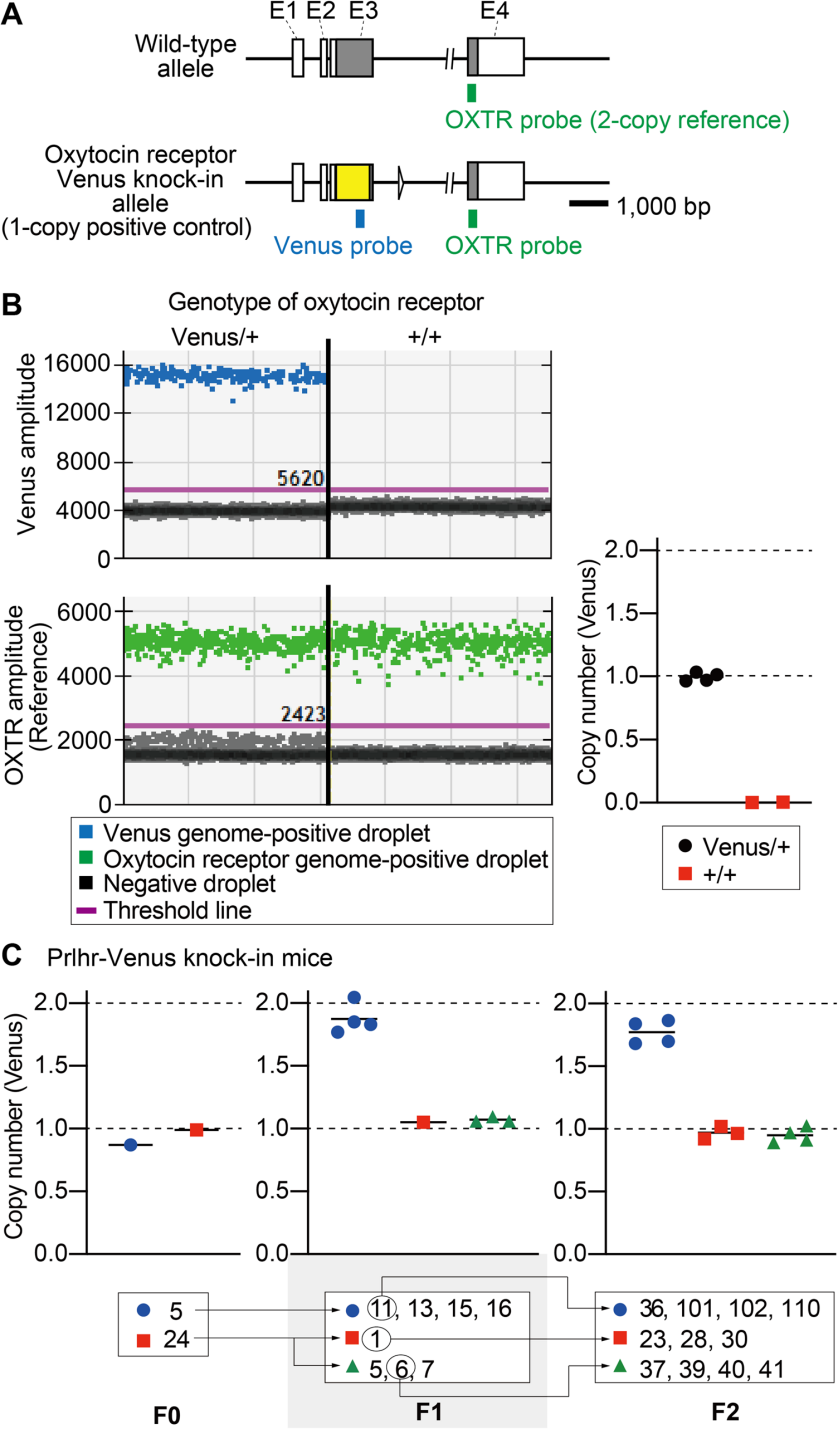
Analysis of Venus gene copy number using droplet digital PCR. (**A**) Oxytocin receptor (OXTR)-Venus knock-in heterozygous mice used as 1-copy positive controls. Schematic representation of the wild-type mouse OXTR genomic locus (Upper) and the genomic locus with Venus knock-in are shown (Lower). Exon 4 (E4) of the OXTR gene was used to normalize the Venus copy number as a 2-copy reference gene. The green and blue bars show specific probe sets for OXTR and Venus, respectively. (**B**) Reliable detection of the Venus gene copy number using genomic DNA from OXTR-Venus knock-in heterozygous mice generated by embryonic stem cells. Representative droplet plots of oxytocin receptor Venus/ + and + / + (Venus-positive droplets [Upper left], OXTR positive-droplet [Lower left]). Calculated Venus gene copy number in oxytocin receptor Venus/ + and + / + mice (right). (**C**) Calculated copy numbers of the Venus gene in the F0, F1, and F2 generations of the Prlhr-Venus knock-in mice. Black arrows show the parent–offspring relationships.

We conducted droplet digital PCR to determine the copy number of the transgene. We first confirmed whether the copy number of the Venus gene was accurately detected using droplet digital PCR. Oxytocin receptor-Venus knock-in heterozygous mice generated by traditional ES cells were used as a positive control for a single copy of the Venus gene (Fig. 2A)^30^. The average copy number of the Venus gene in the oxytocin receptor-Venus knock-in heterozygous and wild-type mice was calculated to be 1.00 and 0.00, respectively (Fig. 2B). These results confirmed that the copy number of Venus gene integration in the mouse genome could be accurately detected.

We then conducted droplet digital PCR for two candidates of the F0 generation. The copy number of the Venus gene in numbers 5 and 24 of the F0 was calculated to be one (left panel of Fig. 2C). In the F1 generation, numbers 1, 5, 6, and 7 were also calculated to have one copy. These results indicate that the offspring of number 24 of the F0 had a copy number similar to that of its parent. In contrast, although number 5 of the F0 had one copy, numbers 11, 13, 15, and 16 had two copies (middle panel of Fig. 2C). The Venus copy number in the F2 was similar to their parents (right panel of Fig. 2C). Based on droplet digital PCR analysis, both of the two F0 generation mice that were candidates by conventional PCR were confirmed to have one copy. The results from the F1 and F2 generations suggest that some germ cells from F0 number 5 had two copies of the transgene and those from F0 number 24 had one insertion copy. This demonstrates that droplet digital PCR analysis using only the tail genome of the F0 generation was unable to accurately estimate copy number in the F1 generation.

### Southern blot analysis

**Figure 3:**
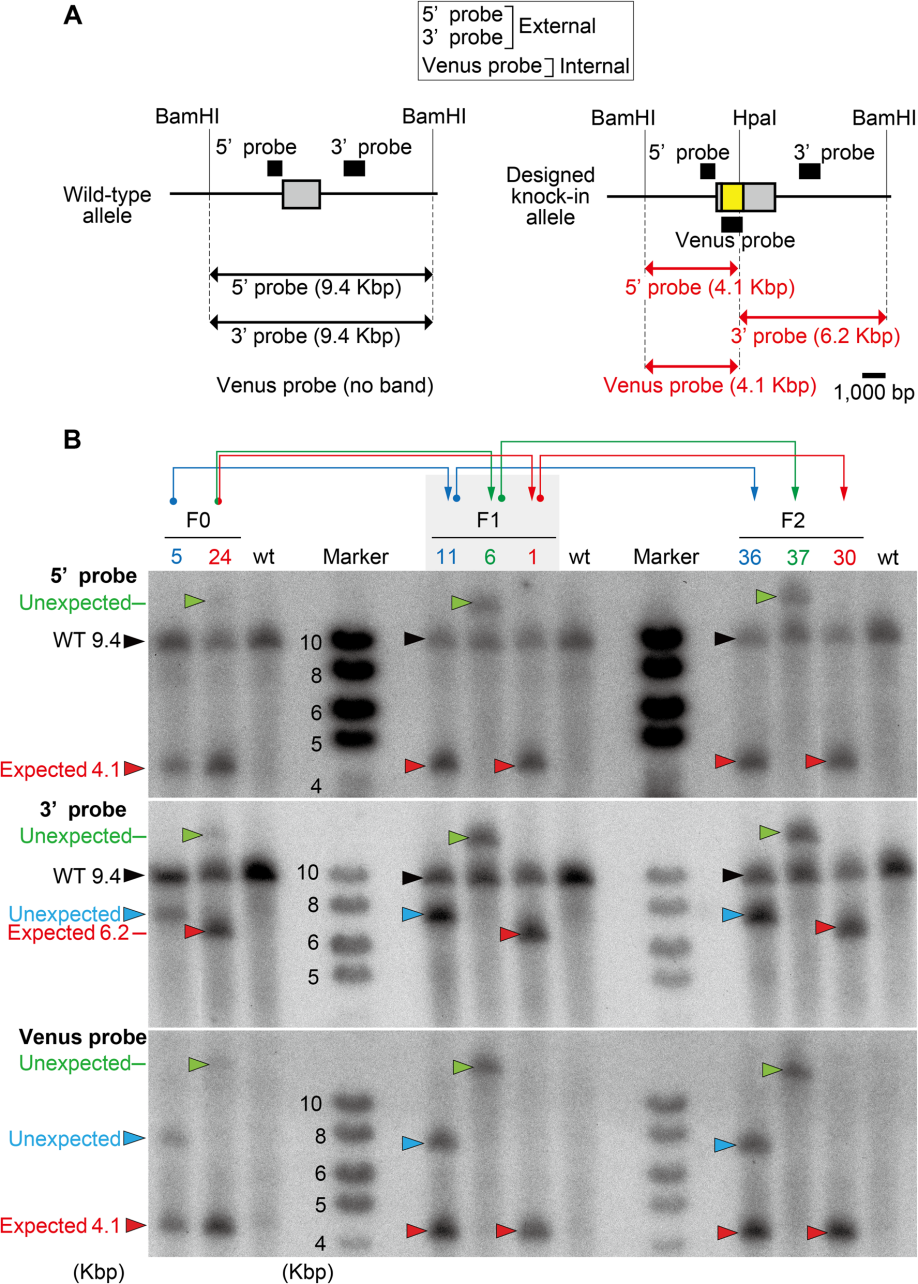
Southern blot analysis of targeted gene recombination. (**A**) Schematic representation of the wild-type mouse Prlhr genomic locus (left) and genomic locus with designed recombination (right). The black bars show the specific probes used for Southern blotting. The horizontal arrows denote the expected sizes of the restriction DNA fragments. (**B**) Southern blot analysis of genomic DNA from the F0, F1, and F2 generations and wild type (wt). Blue, green, and red arrows show the parent–offspring relationships. Black and red arrowheads show the DNA fragment from the wild-type and designed knock-in allele, respectively. Green and blue arrowheads show the DNA fragments from unintentional mutant alleles. Uncropped gel images are presented in Supplemental Fig. 9B.

We performed Southern blot analysis to characterize Prlhr locus-specific targeting. Restriction enzymes BamHI and HpaI were selected from a putative designed knock-in allele for digestion of genomic DNA (Fig. 3A). 5’ and 3’ probes external to the targeting vector and an internal Venus probe were used to distinguish the wild-type (9.4-kbp band for the 5’ and 3’ probes, and no band for the Venus probe) and designed target allele (4.1-kbp for the 5’ and Venus probes and 6.2-Kbp for the 3’ probe; Fig. 3A).

None of the F0 mice showed the designed band pattern by Southern blot analysis. In F0 number 5, the 5' probe detected the expected band (red arrowhead, left upper panel of Fig. 3B), but the 3' probe detected a band that was larger in size than expected (blue arrowhead, left middle panel of Fig. 3B), and the Venus probe detected two bands, one expected and another unexpected (red and blue arrowheads, respectively, left middle panel of Fig. 3B). The F1 and F2 generation progeny of F0 number 5 showed exactly the same band pattern (middle and right panels of Fig. 3B). For F0 number 24, all probes detected two bands of the mutation, one expected and another unexpected (red and green arrowheads, respectively, left panels of Fig. 3B). Based on the conventional PCR results, the progeny of the F1 and F2 generation could be divided into two types: those that showed the expected band pattern for all probes (F1 number 1 and F2 number 30, middle and right panels of Fig. 3B) and those that showed unexpected bands for all probes (F1 number 6 and F2 number 37, middle and right panels of Fig. 3B). Unexpected bands were detected with the 5' and 3' probes designed outside the targeting vector sequence, indicating that an unexpected insertion of the Venus gene occurred between the two BamHI sites in the vicinity of the Prlhr locus.

### Next-generation sequencing-based RAISING analysis

**Figure 4:**
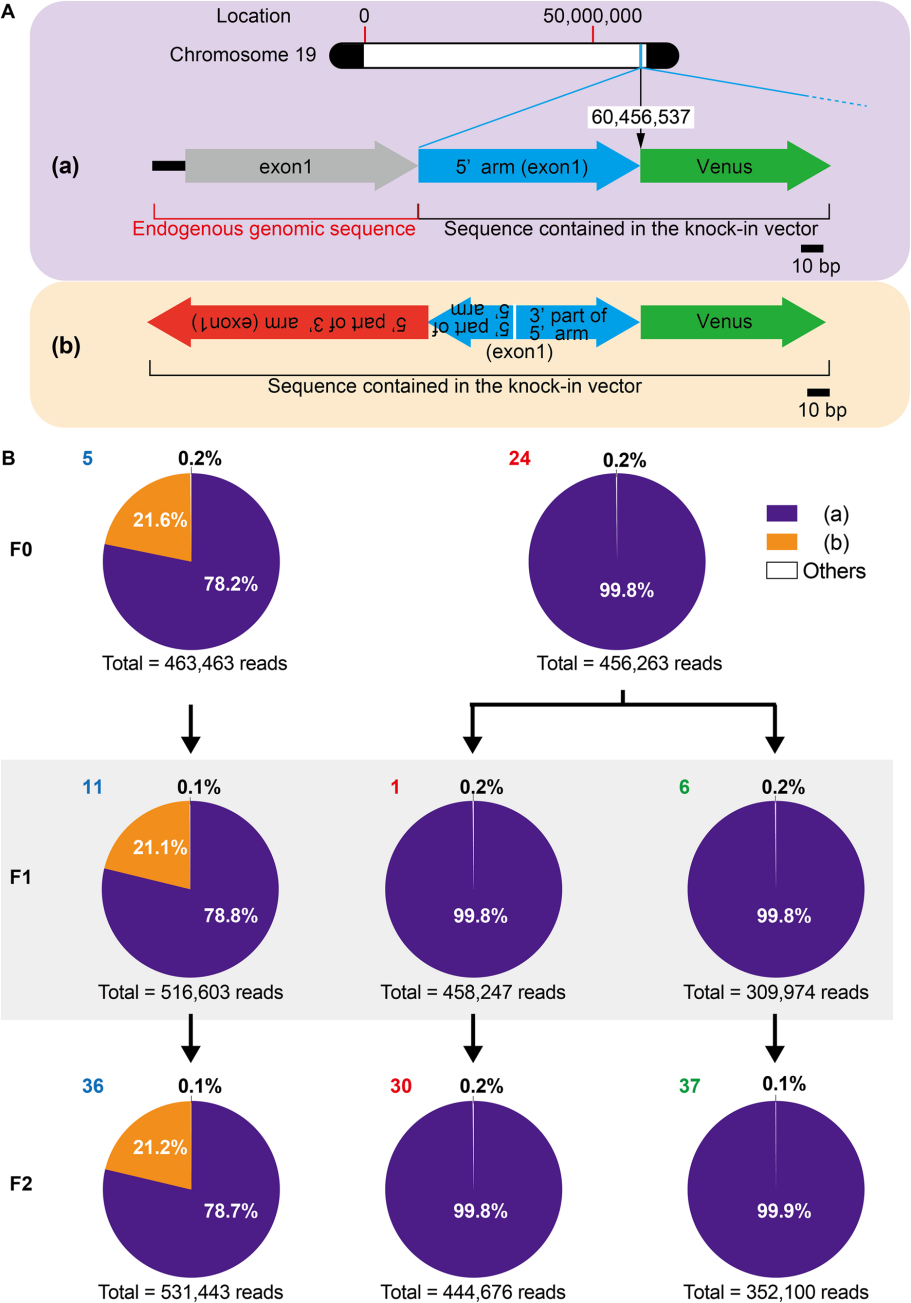
Analysis of the Venus gene integration site using the next-generation sequencing based-RAISING method. (**A**) Schematic representation of two PCR products, type (a) and type (b), containing Venus sequences. The endogenous genomic sequence was included in type (a), and the location of the Venus insertion in the genome was identified as chromosome 19: 60,456,537. Type (b) did not contain endogenous genomic sequences and the location of the Venus insertion was not identified. Type (b) consisted of only the knock-in vector-containing sequence, and parts of the 3' and 5' arms were inverted. (**B**) Proportion of read counts for PCR products containing Venus sequences from the F0, F1, and F2 generations. In F0 number 5 and its progeny, both types (a) and (b) were detected. In F0 number 24 and its progeny, only type (a) was detected. Black arrows show the parent–offspring relationships.

We also performed random integration analysis with next-generation sequencing. This method was developed for sensitive detection of clonality in cells infected with Human T-cell leukemia virus type-1, which causes adult leukemia/lymphoma^31^. In number 5 of the F0 generation, two types of sequences including Venus were detected (Fig. 4A, Supplemental Fig. 1 and Supplemental Table 1). Type (a) contained both the endogenous genomic sequence and the knock-in vector-containing sequence. The Venus integration site was at the designed location of the Prlhr gene on chromosome 19. Type (b) consisted of only the knock-in vector-containing sequence, and parts of the 3' and 5' arms were inverted. Among the total of 463,463 reads, the proportions of type (a) and (b) were 78.2% and 21.6%, respectively (Fig. 4B and Supplemental Table 1). In number 24 of F0, type (a) was detected, and the proportion of type (a) was 99.8% out of a total of 456,263 reads. Results for the F1 generation were similar to those of their parents (Fig. 4B and Supplemental Table 1). Even in the F2 generation, results similar to those of their parents were obtained (Fig. 4B and Supplemental Table 1). By RAISING analysis, two different sequences that included a Venus sequence were detected in F0 number 5. This suggests that a tandem two-copy occurred at one Prlhr locus or that the designed one copy knock-in occurred at one Prlhr locus and an inverted insertion of the targeting vector occurred at the other Prlhr locus. However, these two sequences did not separate in the F1 and F2 generation progeny of F0 number 5, suggesting that insertion of a two-copy tandem at one Prlhr locus had occurred. Although the F1 and F2 generations from F0 number 24 were divided into two strains based on the results of conventional PCR, only type (a) was detected in both strains. These results suggest that F1 number 6 and F2 number 37 have a Prlhr locus in which Venus was inserted and genome rearrangement occurred in the vicinity.

### Genomic sequencing analysis

**Figure 5:**
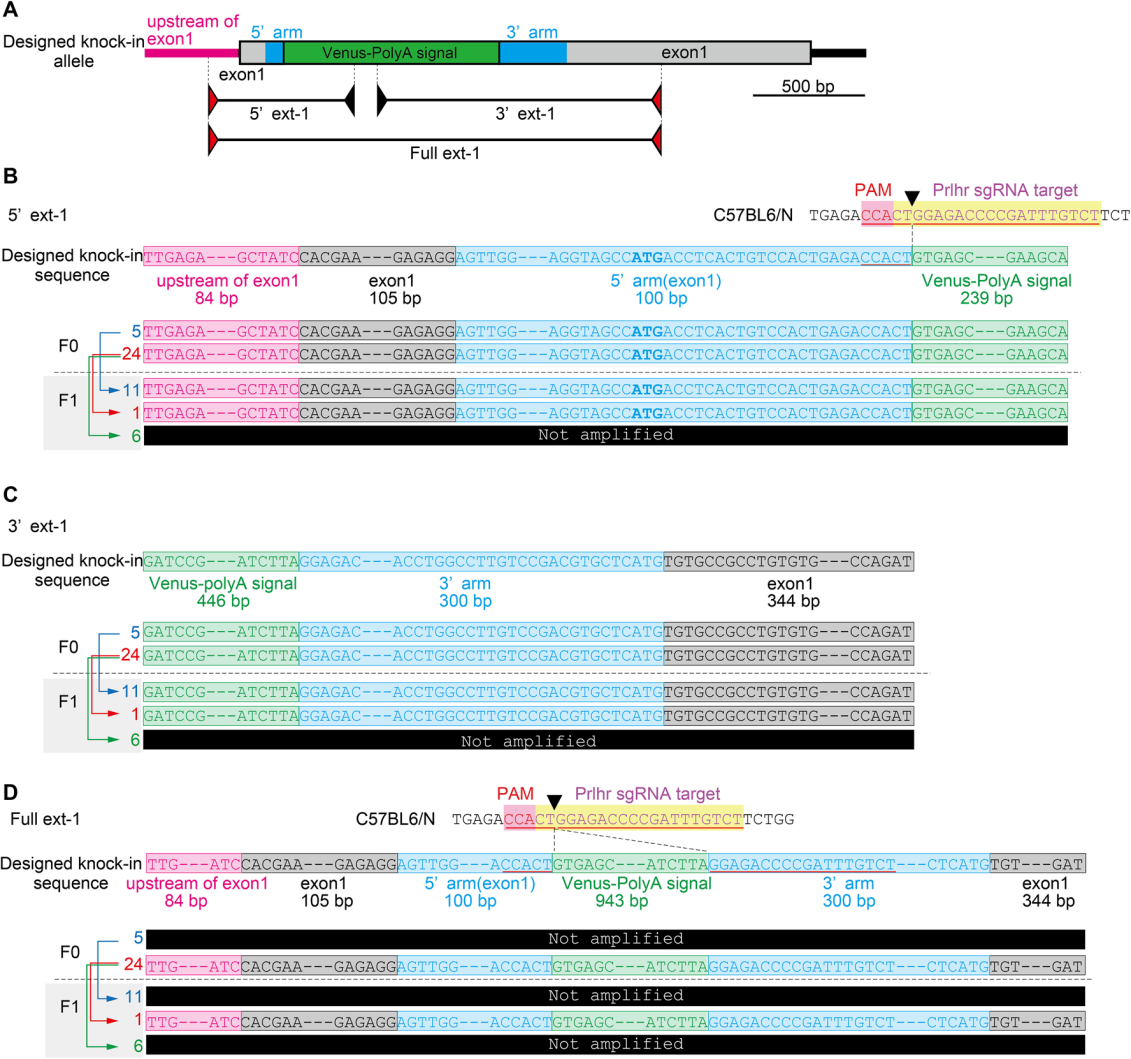
Analysis of genomic sequencing in the F0 and F1 generations. (**A**) Schematic representation of the designed Prlhr knock-in locus and primer sets for PCR amplification (primer external to the targeting vector and internal primer pairs, 5’ext-1 and 3’ext-1; primers external to the targeting vector pair, Full ext-1). Red and black arrows show the primer external to the targeting vector and internal primer, respectively. Sequence analysis for the PCR products of the 5’ ext-1 (**B**), 3’ ext-1 (**C**), and Full ext-1 (**D**). Blue, red, and green arrows show the parent–offspring relationships.

We performed sequence analysis of numbers 5 and 24 of the F0 generation and numbers 11 and 1 of the F1 generation. We used three primer pairs external to the targeting vector and internal primers in combination as shown in Fig. 1. The sequences of the PCR products in the 5' and 3' joint areas were the designed sequences for numbers 5 and 24 of the F0 and their offspring numbers 11 and 1 of the F1 (Fig. 5A–C, Supplemental Figs. 2 and 3). PCR products containing the full length targeting vector were analyzed, and sequences of the designed mutant alleles were detected in number 24 of the F0 and number 1 of the F1 (Fig. 5A, D and Supplemental Fig. 4). In number 1 of the F1, PCR products were also detected by conventional PCR using primer pairs located outside of the primers used for the Full ext-1 as well as the primer located downstream of exon 1 and the internal primer (Supplemental Fig. 5A and B). No unexpected mutations in the confirmed range from exon 1 upstream to downstream of the knock-in allele were detected by sequence analysis of the PCR products (Supplemental Figs. 5C,D, 6 and 7). These results indicated that two candidate mice of the F0 generation had Venus inserted in the Prlhr locus and that each sequence was correctly transmitted to their offspring.

## Discussion

In this study, we conducted a detailed analysis of the process for generating a Prlhr-Venus knock-in mouse line using five genotyping methods spanning three generations. After the comprehensive analysis, we found that one F0 generation mouse had the designed knock-in allele, and we were able to establish a knock-in mouse line with that designed mutant. Number 5 of the F0 had a tandem two-copy mutant allele. F0 number 24 was a mosaic mouse with the designed mutant allele and an allele that underwent reconstruction near the Prlhr locus with the targeted knock-in. These two mutant alleles were separated in the F1 generation.

### Validation of conventional PCR

In conventional PCR, the use of primers external to the targeting vector is effective because they can confirm knock-ins to the target locus. However, in the present study PCR products from the 5' and 3' regions were detected using the external and internal primer pairs even in the two-copy mutant, suggesting that conventional PCR alone did not guarantee a designed one-copy mutant. The multicopy event occurred with the one guide RNA-one donor DNA method as well as in the previously reported conditional knockout method using two guide RNAs-one donor DNA containing two loxP sequences^23^. In this study, PCR products containing the full length target vector were detected in mice with a one-copy mutant allele using external primer pairs. The detection of this PCR product seems to be the most reliable method for detecting a one-copy mutant allele using conventional PCR. However, in a different study on the generation of a conditional knock-out using the two guide RNA-one lssDNA method, no PCR products were detected using external primer pairs despite the designed one-copy integration^23^. In addition, in the majority of studies on improved knock-in methods using CRISPR-cas9, it was not determined whether PCR products could be detected or not using external primer pairs^14–17,19–21^, although the correct product was reported in one paper^18^. PCR amplification with external primer pairs is important for confirmation of a single copy of the transgene, although the true capability of PCR amplification may depend on the characteristics of the targeting vector and the target locus. Thus, just because the product is not detected does not necessarily mean that the expected mutation did not occur.

For example, number 24 of the F0 was a mosaic genotype with two different knock-in mutant Prlhr alleles. This mosaicism was not detected by conventional PCR using the genome of number 24 of the F0, and could only be detected using multiple primer pairs for the genomes of the F1 and F2 generations. Conventional PCR is most often used for genotyping for strain maintenance. However, at least for the F1 generation, multiple external primer pairs should be used to select rodents with the designed mutation and to remove unexpected mutants.

### Validation of droplet digital PCR

**Figure 6:**
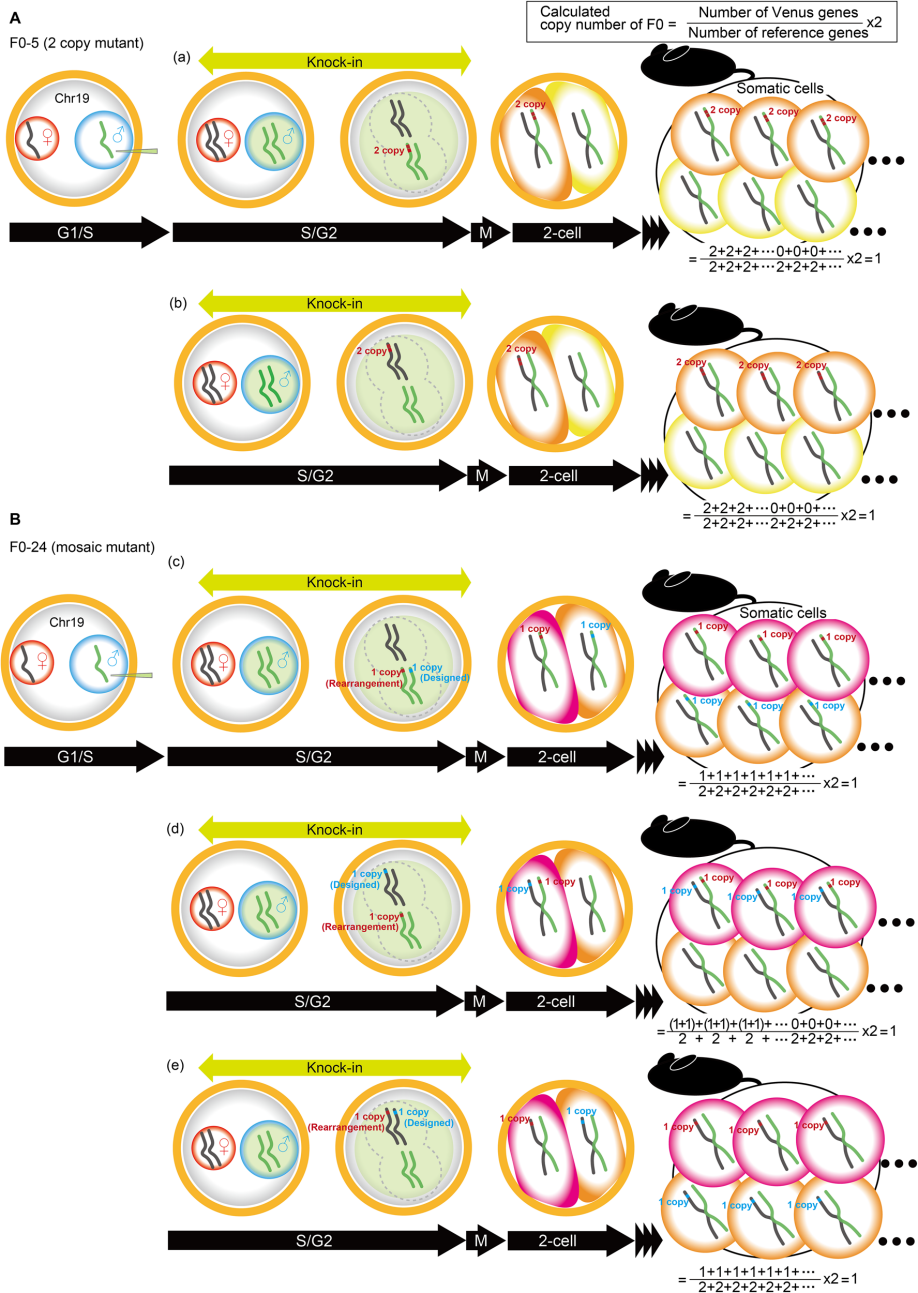
Schematic drawing of the estimated knock-in events that occurred at the Prlhr locus in the F0 generation. A cocktail of hCas9 mRNA, sgRNA, and lssDNA was injected into the male pronuclei of embryos. HDR-mediated knock-in events occur in the S/G2 phase of the pronuclear stage. (**A**) In F0 number 5, tandem two-copy insertions occurred on (a) one paternal or (b) one maternal chromosome. Based on droplet digital PCR using the genome extracted from the tail of F0 number 5, copy number was calculated to be a single copy mutant in either case. (**B**) In F0 number 24, single-copy insertions occurred in two different Prlhr loci, one KI allele was present as designed and the other was an allele in which rearrangement occurred: (c) two paternal chromosomes, (d) one paternal chromosome and one maternal chromosome, or (e) two maternal chromosomes. By droplet digital PCR using the genome extracted from the tail of F0 number 24, copy number was also calculated to be a single copy mutant in all cases.

We first confirmed the accuracy of copy number detection by droplet digital PCR using oxytocin receptor-Venus knock-in heterozygous mice that were established using ES cells via homologous recombination. In these mice, copy number analysis for the Venus gene was able to detect the one-copy mutant with high confidence. In the Prlhr-Venus knock-in mice, number 5 of the F0 generation was identified as a one-copy mutant. Conversely, all heterozygous progeny from number 5 of the F0 were identified as two-copy mutants over the F2 generation. HDR-mediated repair occurs during the S and G2 phases, when sister chromatids are formed^32^. Thus, four Prlhr loci can temporarily exist in a one-cell fertilized egg. In the case of number 5 of the F0, the two targeting vectors were inserted into one locus in tandem, and thus when droplet digital PCR was performed on the genomic DNA extracted from the F0 mouse, it was identified as having only one copy (Fig. 6A). Number 24 of the F0 was identified as a one-copy mutant and all heterozygous progeny of that mouse were also detected as one-copy mutants. In this case, one targeting vector was inserted into two of the four loci; this resulted in the droplet digital PCR calculating it as only one copy (Fig. 6B).

For the F1 and F2 heterozygous mice, the copy number obtained by droplet digital PCR is quite reliable because the knock-in allele and the wild-type allele are present in a one-to-one ratio (Supplemental Fig. 8). Droplet digital PCR is not necessary for the F0 mice because copy number cannot be accurately calculated due to the potential for multi-copy or mosaic genotypes. However, estimation of knock-in events occurring in F0 embryos, performed by comparing copy number results in the F0 and F1 with droplet digital PCR, is valuable for the development of efficient and appropriate knock-in methods.

### Validation of southern blotting

Although Southern blotting is a classic method, we were able to detect both designed and unexpected mutant loci. In order to obtain sufficient information from the genomic DNA of the F0 generation, it was necessary to use all 5’ and 3’ probes external to the targeting vector and internal Venus probe. In number 5 of the F0, the band pattern was detected as expected in the 5' probe. However, the band detected with the 3' probe was larger than expected, and the Venus probe also detected a band other than the expected. These results suggested that knock-in occurred at the Prlhr locus but with an unexpected multicopy mutation, which could not be detected by the 5' probe alone. For F0 number 24, all probes detected both unexpected and expected bands. This suggests that the mice had two different mutations at the Prlhr loci. Based on the PCR results, the progeny were divided into two genotypes, and it was determined that the F0 had a mosaic of two knock-in genotypes. A larger band than that of the wild type was detected using the external probes, thus confirming that rearrangement occurred in the vicinity of the Prlhr locus.

Southern blotting in the F1 generation should be performed to confirm the presence of the designed mutant. External probes can detect gross genomic changes near the target locus, while internal probes can distinguish integration events including off-target insertions from the entire genome. The weakness of Southern blotting is that it cannot detect small insertion or deletion mutations around the target locus, which often occur with the CRISPR-Cas9 method^15,21^, if they occur at the same time as the knock-in event to the target locus.

### Validation of next-generation sequencing-based RAISING

The RAISING method was used in the present study to detect knock-ins within the target locus and off-target insertions. Two different sequences were detected for number 5 of the F0 generation. Type (a) contained sequences outside the targeting vector and showed the designed knock-in. Type (b) contained sequences with partially inverted 5' and 3' homology arms but did not include genomic sequences outside the targeting vector. This result was attributed to either a tandem two-copy at one Prlhr locus or a designed one-copy knock-in at one Prlhr locus and a reverse insertion of the targeting vector at the other Prlhr locus. Southern blot results for F0 number 5 showed that the designed knock-in-derived band was not obtained with the 3' probe, suggesting that a tandem two-copy likely occurred. Both types of sequences were also detected in the progeny of number 5 from the F0, and the proportions of their sequence reads were the same as in the F0. The fact that type (a) and type (b) did not separate in the F1 and F2, that the sequence of type (b) contained the sequence of the 3' arm region, and that all heterozygous progeny of number 5 of the F0 were also identified as two-copy mutants by droplet digital PCR suggested that the sequences containing these two Venus genes were arranged in tandem at the Prlhr locus.

In number 24 of the F0, only the type (a) sequence was detected. Based on results from conventional PCR, the strain was divided into two groups, but only type (a) was detected in all F1 and F2 mice. Number 6 of the F1 and number 37 of the F2 were predicted to have off-target insertions because no PCR products were detected by conventional PCR using external primers. However, combined with the Southern blot results, in which the mutant allele was detected with the 5' and 3' probes, the targeted knock-in was found at the Prlhr locus but with reconstruction occurring in the vicinity. Unlike conventional PCR for which results depend on the selection of primer pairs, the RAISING method provides information on the insertion of exogenous gene sequences throughout the entire genome at the sequencing level. The complexity of the data analysis is expected to be much simpler than whole genome sequencing using a next-generation sequencer because only the region associated with the insertion of mutations is sequenced. Interpretation of unexpected bands due to two-copy mutants in Southern blotting of the F0 generation is easier when combined with sequences from the RAISING method. However, the weakness is that the maximum sequence length per read is 300 bp, and unless the homologous arm sequence is less than 250 bp, the sequence cannot reach the endogenous genomic sequence and the insertion position cannot be identified. Moreover, while it is less expensive than whole genome sequencing, it is more expensive than conventional sequencing. In the F1 generation where mosaicism is resolved, droplet digital PCR and genomic sequencing are more useful for copy number detection and sequence confirmation.

### Validation of genomic sequencing

Sequence confirmation is particularly important for the CRISPR-Cas9 method which can induce insertion and deletion mutations. These mutations can occur in the junction region along with the knock-in of the targeting vector^15,21^. In the PCR products of the 5' and 3' regions using external primers, number 5 of the F0 generation and its offspring, number 11 of the F1, showed all the designed junction sequences. These results indicate that correct junctions were generated at the 5’ and 3’ parts in tandem multicopy mutants and that even if the sequences of the 5’ and 3’ junctions are correct, they cannot guarantee a single copy mutant. In number 24 of the F0, the PCR product containing the full length of the targeting vector using external primers was confirmed to be the designed sequence. In the present study, lssDNA was used as the donor DNA, and no unexpected mutations were found in any of the sequences analyzed, including the Venus-polyA signal cassette. However, in a previous study using two gRNAs and one lssDNA to generate conditional knock-out mice, unexpected point mutations occurred in the sequence within the targeting vector^22^. Thus, it is necessary to verify the sequence within the targeting vector as well as the sequence at the 5' and 3' junctions in the F1 generation.

### Four possible knock-in events using the CRISPR-cas9 method

**Figure 7:**
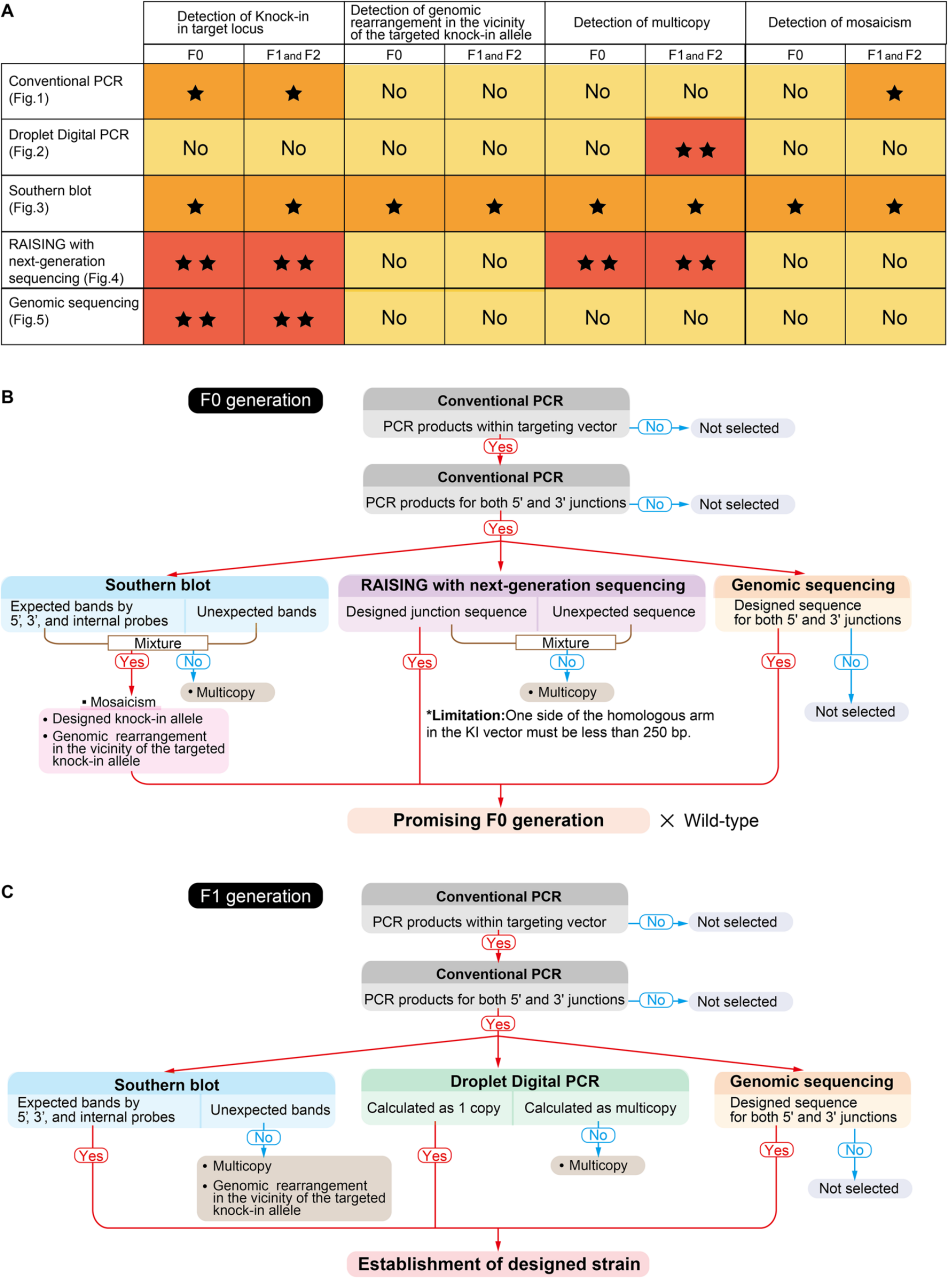
Comparison of detection performance amongst the five analytical methods for four possible knock-in events using CRISPR-cas9, the selection process for a promising F0 generation, and the designed strain in this study. (**A**) Based on results obtained from this study, the superiority of each method for detecting the mutation events is summarized. **More informative, *informative, and ^No^ Not informative or least information. (**B**) The flow for sorting a promising F0 generation is illustrated. Southern blotting was able to detect mosaicism and multicopies. The RAISING method was also capable of detecting multicopies. Conventional PCR and genome sequencing were unable to detect mosaicism and multicopies. (**C**) The flow for establishing a designed strain in the F1 generation is illustrated. Southern blotting identified multicopies and genomic rearrangements near the target knock-in allele. Droplet digital PCR could distinguish multicopies. Conventional PCR and genome sequencing were unable to detect multicopies.

In the generation of knock-in mice by homologous recombination of ES cells, chimeric mice can be obtained using a single clone with the correct recombination selected from a large number of ES cell clones. Therefore, chimeric mice of the F0 generation have a unitary knock-in allele. The knock-in allele of the F1 mice obtained by crossing chimeric mice with wild type is also identical to that of the injected ES cells. However, careful analysis is required for F0 mice obtained by the CRISPR-cas9-based knock-in method, because the somatic genomic DNA is a mixture of designed and unexpected mutations. In order to select promising F0 generations and establish designed strains, we used five genome analysis methods to detect knock-ins at the target locus, genomic rearrangements in the vicinity of the knock-in allele, and multicopy and mosaic genotypes (Fig. 7A). For detection of knock-ins within the target locus, conventional PCR and Southern blot analysis provided accurate information, the RAISING method and genome sequencing were then particularly informative because they provided sequence data. This was similar for both the F0 generation and the F1 and F2 generations. Genomic rearrangements occurring in the vicinity of the knock-in allele could only be detected by Southern blotting, and this was similar for the F0, F1, and F2 generations. For detection of multicopies, Southern blotting and the RAISING method were effective in the F0 generation, while in the F1 and F2 generations, in addition to these methods, the increased accuracy from droplet digital PCR was useful. Mosaicism was detected by Southern blotting in the F0 generation, whereas in the F1 and F2 generations, mosaicism was eliminated and consequently could also be identified by conventional PCR.

## Conclusion

Candidate selection should be made based on the assumption that F0 generation mice generated by the CRISPR-cas9-based knock-in method contain a mixture of designed and unexpected mutations. Analytical methods that can reveal both unexpected and designed mutations enable a more confident selection of promising F0 mice. Southern blotting was particularly useful for detecting unexpected mutations in the whole genome when all external 5', 3', and internal probes were used. Interpretation of unexpected bands due to multicopy variants by Southern blotting was easier when combined with sequences from the RAISING method, although the RAISING method is limited in that one side of the homologous arm in the knock-in vector must be less than 250 bp. For the F0 generation, the combination of four methods, conventional PCR, Southern blotting, RAISING, and genome sequencing, was very effective (Fig. 7B). Moreover, this series of analyses can be completed before the F0 generations become fertile. For the F1 generation, the results of droplet digital PCR in addition to conventional PCR, Southern blotting, and genome sequencing, were beneficial for establishing the designed strain (Fig. 7C). A combination of these methods is sufficient, and the RAISING method was not necessary in this case. On the other hand, in the case of a tandem multicopy, regardless of the generation, conventional PCR, Southern blotting, and sequencing yielded results that were partially identical to those of a one-copy insertion. These results demonstrate that focusing only on positive evidence can lead to erroneous selection of undesigned strains. Of course, if we can determine whether additional F0 mice should be obtained without waiting for analysis of the F1 generation, a great amount time and effort can be saved. The most important consideration, however, is to establish a strain with the designed knock-in mutation. Careful analysis of the F1 generation, which eliminates mosaicism, is essential to this goal.

## Materials and methods

### Animals

Mice of the C57BL/6 N strain (Charles River Laboratories, Kanagawa, Japan) and oxytocin receptor-Venus knock-in heterozygous mice backcrossed with C57BL/6 J mice for over 18 generations^30^ were used in the present study. The mice were housed under a 12:12‐hour light/dark photocycle (lights on 7:30 am) at 20–24 °C and 40–70% relative humidity. Food and water were available ad libitum. All animal procedures were approved by the Institutional Animal Experiment Committee of Jichi Medical University and Institute of Immunology Co., Ltd. and were conducted in accordance with the Institutional Regulations for Animal Experiments and Fundamental Guidelines for Proper Conduct of Animal Experiments and Related Activities in Academic Research Institutions under the jurisdiction of the Ministry of Education, Culture, Sports, Science and Technology. Animal experiments were performed in accordance with the “Animal Research: Reporting of In Vivo Experiments” (ARRIVE) guidelines (https://www.nc3rs.org.uk/arrive-guidelines).

### Preparation of Cas9 mRNA and sgRNA

Expression vectors for hCas9 containing the T7 promoter, SV40 nuclear localization signal-fused hCas9, and an 81-bp polyadenylation signal were used^15^. Cas9 mRNA was transcribed in vitro from a linearized plasmid, a poly(A) tail was added and purified using the MessageMAX T7 ARCA Capped Message Transcription Kit (Cellscript, LLC., Wisconsin, USA), poly(A) Polymerase Tailing Kit (Epicentre Biotechnologies, Wisconsin, USA), and MEGAClear Kit (Thermo Fisher scientific, Inc., Massachusetts, USA). Software tools for sgRNA design, CRISPR design^33^, and CRISPR direct (RRID:SCR_018186) were used for prediction of unique target sites throughout the mouse genome. Synthesis and purification of sgRNA were performed by FASMAC Co., Ltd (Kanagawa, Japan).

### Preparation of lssDNA

A DNA fragment containing a Venus-SV40 polyadenylation signal cassette and two homology arms was cloned into a pUC plasmid. A 100-bp fragment was used as the 5’ homology arm and a 300-bp fragment was used as the 3’ homology arm (Supplemental Table 2). The target sequence in the plasmid was amplified by PCR with a primer pair containing a nuclease resistant primer and the PCR product was digested with 5'–3' exonuclease to produce lssDNA. After digesting the template plasmid, the lssDNA was sequenced and confirmed to be lssDNA by capillary electrophoresis. The lssDNA was stored at − 80 ℃ until use.

### Microinjections into mouse embryos

Female mice were superovulated by injection of pregnant mare serum gonadotropin (PMSG, ASKA Pharmaceutical Holdings Co., Ltd., Tokyo. Japan) and human chorionic gonadotropin (hCG, ASKA Pharmaceutical Holdings Co., Ltd.). Pronuclear-stage embryos were then collected from the superovulated females. The embryos were cultured in KSOM medium (ARK Resource, Kumamoto, Japan) before and after microinjections. A mixture of 200 ng/mL Cas9 mRNA, 100 ng/mL sgRNA, and 50 ng/mL lssDNA was microinjected into the male pronuclei of embryos using a micromanipulator (Narishige, Tokyo, Japan). Swelling of the pronuclei due to the injection (approximately 1–2 pL) was used as a confirmation of successful injection^34^. The embryos were cultured in KSOM medium then transferred into pseudopregnant female mice.

### Conventional PCR and sequencing analysis for detection of genomic mutations

Approximately 10 ng of genomic DNA extracted from the tail was used. Genomic PCR was performed in a 25-mL reaction volume containing HotStarTaq DNA polymerase (Qiagen, Hilden, Germany) or Q5 High-Fidelity DNA polymerase (New England Biolabs), genomic DNA, and 12.5 pmol of each primer. The primers used are shown in Supplemental Table 3. Oxytocin receptor-Venus knock-in heterozygous and wild-type mice were used as positive and negative controls, respectively. PCR products were directly sequenced using the BigDye terminator v3.1 and the Applied Biosystems 3130xl DNA Sequencer (Thermo Fisher Scientific, Inc.) according to the manufacturer's standard protocol.

### Southern blot analysis for detection of mutants

Five µg of genomic DNA extracted from the tail was digested with BamHI and HpaI (New England Biolabs, Massachusetts, USA) and loaded on 0.8% agarose gels. The digested DNA samples were subjected to electrophoresis and transferred to Hybond-XL membranes (Cytiva, Tokyo, Japan). The membranes were hybridized to ^32^P-labeled DNA probes. The probes were obtained by digestion with restriction enzymes and labeled with DNA polymerase I, Large (Klenow) Fragment (New England Biolabs) and random primers (Takara Bio Inc., Shiga, Japan) with [^32^P]dCTP (PerkinElmer, Massachusetts, USA). The probes used are shown in Supplemental Table 4.

### Droplet digital PCR for determination of transgene copy number

Droplet digital PCR was performed using a QX200 droplet digital PCR system (Bio-Rad Laboratories, Inc., California, USA). Genomic DNA extracted from the tail was digested with the restriction enzyme TaqαI (New England Biolabs). The mouse oxytocin receptor gene was used as a reference gene for normalization of Venus copy number. The assay was performed in a 20-µL reaction volume containing 2 ng of digested genomic DNA, ddPCR Supermix for Probes (Bio-Rad Laboratories, Inc.), gene-specific primers, and hydrolysis probes. Each reaction was performed in duplicate. The hydrolysis probe sets used are shown in Supplemental Table 3. The hydrolysis probe set for the mouse oxytocin receptor gene was designed in exon 4. Oxytocin receptor-Venus knock-in heterozygous mice generated by embryonic stem cells were used as one-copy positive controls. In these mice, part of exon 3 was replaced with a Venus-polyadenylated signal cassette, but exon 4 is intact. Droplet digital PCR data were analyzed with QuantaSoft version 1.7 software (Bio-Rad Laboratories, Inc.), and the number of Venus gene copies was calculated using the OXTR gene as a 2-copy reference per genome.

### Next-generation sequencing

Rapid amplification of integration sites was performed according to a previous report with minor modifications^31^. Genomic DNA extracted from the tail was used. Specific primers used for the amplification in this study are shown in Supplemental Table 3. The final PCR products were purified using the Agencourt AMPure XP kit (Beckman Coulter, California, USA) and were quantified with a Qubit dsDNA HS assay kit (Thermo Fisher Scientific, Inc.) and an Agilent BioAnalyzer with a High-Sensitivity DNA chip (Agilent Technologies, California, USA). Next-generation sequencing was performed using the MiSeq Reagent Kit v3 (600-cycle) on the Illumina MiSeq system (Illumina, California, USA) according to the manufacturer’s protocols.

For data analysis, amplicon-sequence reads of less than 50 nucleotides and low-quality sequencing reads were excluded using fastp software^35^ (Supplemental Table 5). Adapter sequences were also trimmed with fastp (RRID:SCR_016962). A homology search was then performed using Magic-BLAST (RRID:SCR_015513), and trimmed sequence reads that had a sequence of both 20 or more nucleotides of Venus and 90% or greater match identity with the *Mus musculus* genome were extracted. Genomic locations of Venus were determined on the *Mus musculus* genome GRCm39 for all extracted sequence reads. The extracted sequence reads were grouped on the basis of Venus insertion sites and were analyzed with SnpEff software (RRID:SCR_005191) which annotates functional effect prediction (Supplemental Table 1).

## Supplementary Information

Supplementary Legends.

Supplementary Figure S1.

Supplementary Figure S2.

Supplementary Figure S3.

Supplementary Figure S4.

Supplementary Figure S5.

Supplementary Figure S6.

Supplementary Figure S7.

Supplementary Figure S8.

Supplementary Figure S9.

Supplementary Table S1.

Supplementary Table S2.

Supplementary Table S3.

Supplementary Table S4.

Supplementary Table S5.

## Data Availability

The raw reads of next-generation sequencing in this study are available from the DDBJ/EMBL/NCBI Sequence Read Archives under the accession number DRA014567. The data supporting the findings of this study are available from the corresponding author upon request.
