# Supplementary material for: Necessity of integrated genomic analysis to establish a designed knock-in mouse from CRISPR-Cas9-induced mutants
Source: Sci Rep. 2022 Nov 27;12:20390. doi: 10.1038/s41598-022-24810-5 (PMC9701781; doi:10.1038/s41598-022-24810-5)
Supplement: Supplementary file 1 — Supplementary Legends. [file 41598_2022_24810_MOESM1_ESM.docx]

**Legends in Supplemental Figures and Tables**

Supplemental Fig. 1. Two types of sequences including the Venus sequence detected by RAISING analysis.

The two PCR products, type (a) and type (b), containing Venus sequences are shown.

Supplemental Fig. 2. Comparison of the 5' ext-1 PCR product of the target genomic region with the designed knock-in sequence.

The designed knock-in sequence was compared to the PCR product of the 5' ext-1 amplified from numbers 5 and 24 of the F0 generation and numbers 11 and 1 of the F1 generation. No unexpected mutations were detected in any PCR products.

Supplemental Fig. 3. Comparison of the 3' ext-1 PCR product of the target genomic region with the designed knock-in sequence.

The designed knock-in sequence was compared to the 3' ext-1 PCR product amplified from numbers 5 and 24 of the F0 generation and numbers 11 and 1 of the F1 generation. No unexpected mutations were detected in any PCR products.

Supplemental Fig. 4. Comparison of the Full ext-1 PCR product of the target genomic region with the designed knock-in sequence.

The designed knock-in sequence was compared to the Full ext-1 PCR product amplified from number 24 of the F0 generation and number 1 of the F1 generation. No unexpected mutations were detected in any PCR products.

Supplemental Fig. 5. Analysis of conventional PCR and genomic sequencing in number 1 of the F1 generation.

(A) Schematic representation of the designed Prlhr knock-in locus and primer sets for PCR amplification (primer external to the targeting vector pairs, Full ext-2 and -3; internal primer and primer external to the targeting vector pair, 3’ ext-2). Red and black arrows show the primer external to the targeting vector and internal primer, respectively. (B) Conventional PCR analysis of genomic DNA from number 1 of the F1 generation using each primer pair. Uncropped gel images are presented in Supplemental Fig. 9C. Sequence analysis for PCR products of the Full ext-2 and -3 (C) and 3’ ext-2 (D).

Supplemental Fig. 6. Comparison of the Full ext-2 and -3 PCR products of the target genomic region with the designed knock-in sequence.

The designed knock-in sequence was compared to the Full ext-2 and -3 PCR products amplified from number 1 of the F1 generation. No unexpected mutations were detected in the two PCR products.

Supplemental Fig. 7. The designed knock-in sequence was compared to the 3’ ext-2 PCR product amplified from number 1 of the F1 generation. No unexpected mutations were detected in the two PCR products.

Supplemental Fig. 8. Schematic diagram comparing the calculated copy numbers for the F0 generation and the F1 generation obtained by crossing F0 with the wild type.

(A) Female F0 number 5 had an allele with a tandem two-copy insertion. The egg cell with the two-copy knock-in allele was fertilized by a wild-type sperm and the F1 generation was born. In F1 mice with two-copy alleles, somatic cells were homogeneous and droplet digital PCR correctly detected them as being two-copy. (B) Female F0 number 24 was a mosaic of a one-copy knock-in allele as designed and a one-copy knock-in allele in which genomic rearrangement occurred near the Prlhr allele. Egg cells with one copy each of the knock-in allele were fertilized with wild-type sperm to produce the F1 generation. In F1 mice with one-copy alleles, somatic cells were homogeneous and droplet digital PCR correctly detected them as being one-copy.

Supplemental Fig. 9. Uncropped images of agarose gels and southern blots. (A) Gel images of Fig. 1D. (B) Blot images of Fig. 3B. (C) Gel images of Supplemental Fig. 5B.

Supplemental Table 1. Results from Magic-BLAST mapping and annotation with SnpEff.

Supplemental Table 2. sgRNA designed to target the mouse Prlhr locus and sequence of donor DNA.

Supplemental Table 3. List of oligonucleotides used in conventional PCR, droplet digital PCR and RAISING.

Supplemental Table 4. List of DNA probes used in Southern blot analysis.

Supplemental Table 5. Number of output reads from MiSeq and number of reads after fastp processing.
