# Supplementary figures and images for: Necessity of integrated genomic analysis to establish a designed knock-in mouse from CRISPR-Cas9-induced mutants
Source: Sci Rep. 2022 Nov 27;12:20390. doi: 10.1038/s41598-022-24810-5 (PMC9701781; doi:10.1038/s41598-022-24810-5)

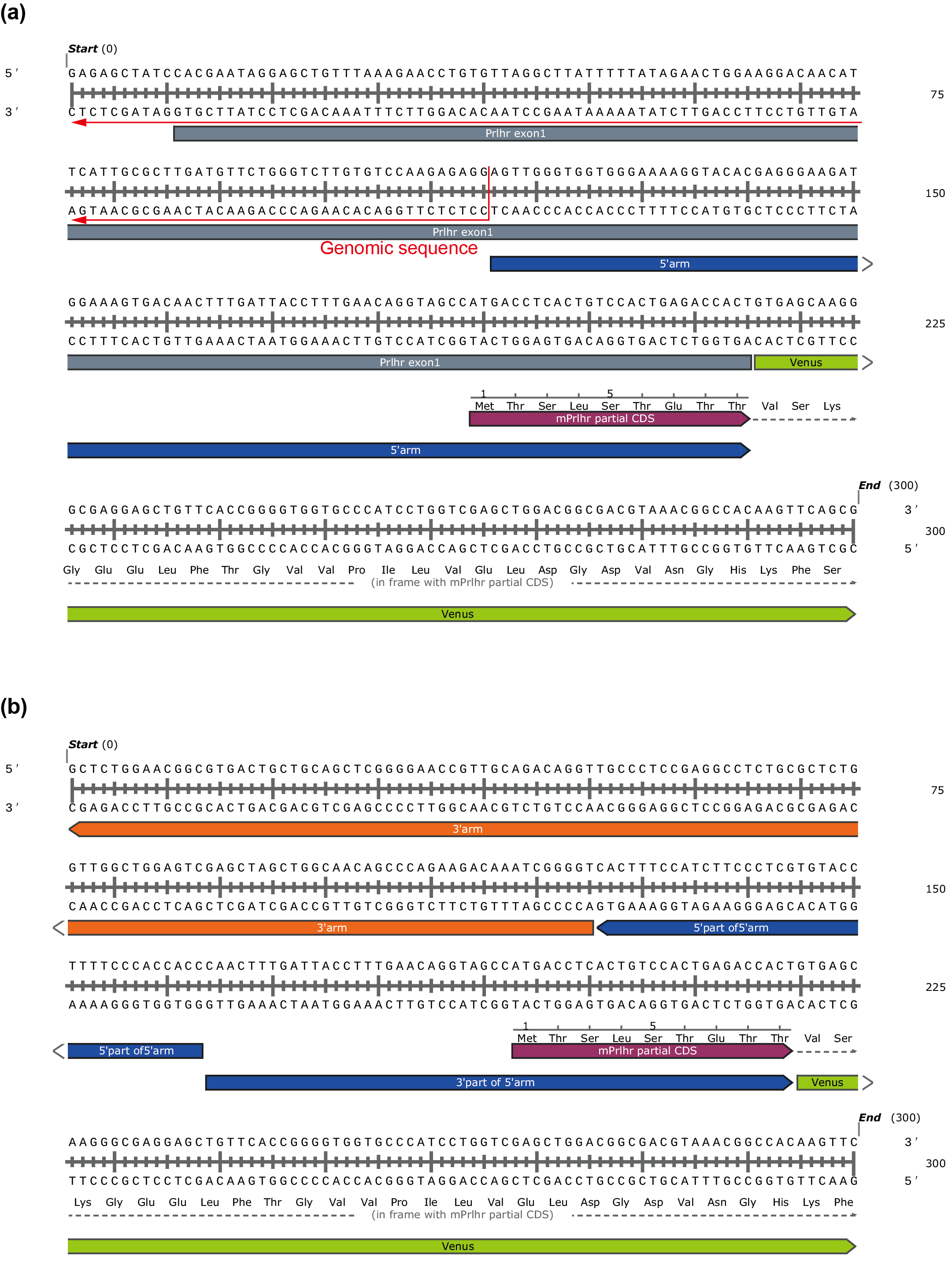

Supplement: Supplementary file 2 — Supplementary Figure S1. [file 41598_2022_24810_MOESM2_ESM.tif]

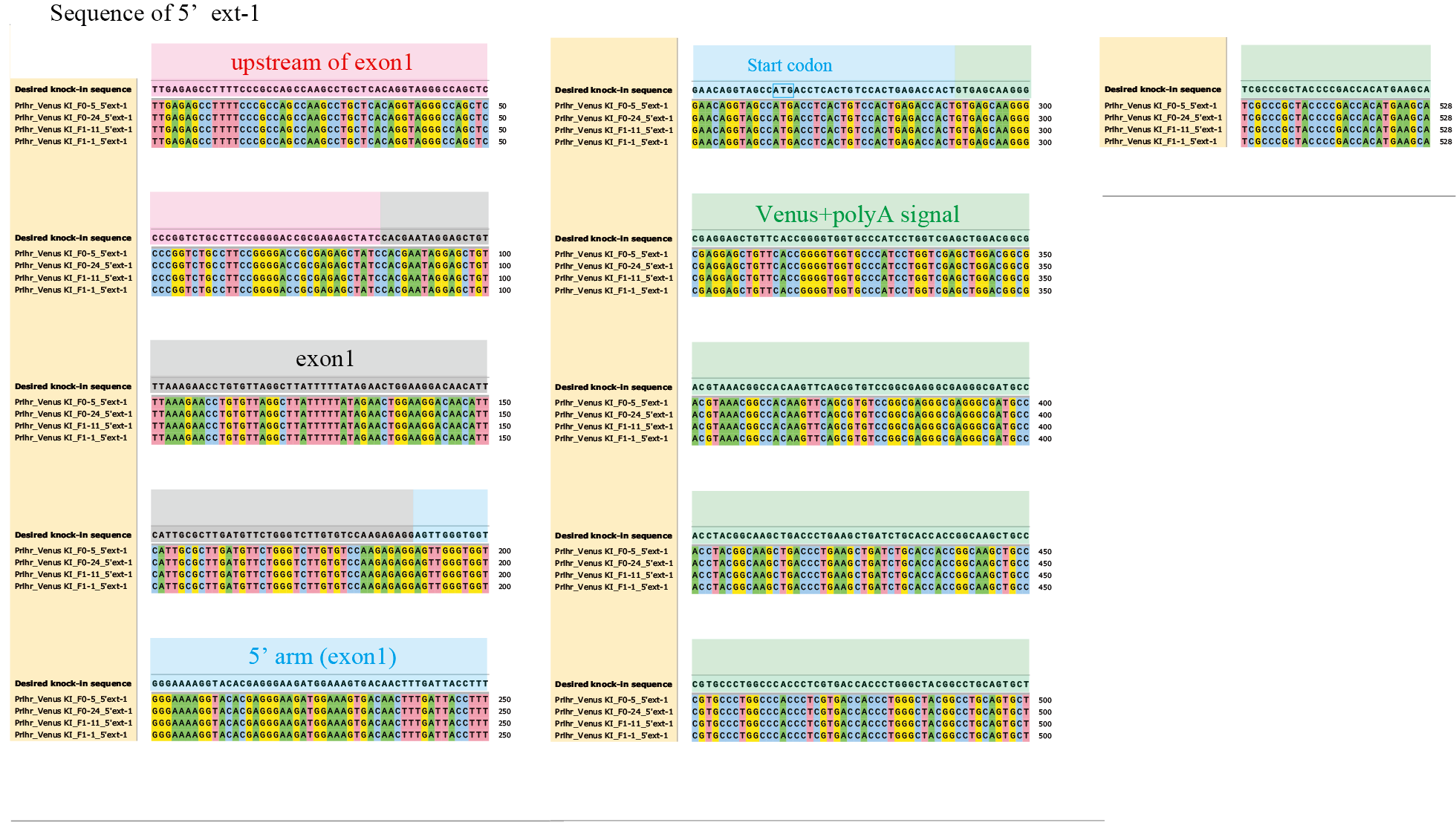

Supplement: Supplementary file 3 — Supplementary Figure S2. [file 41598_2022_24810_MOESM3_ESM.tif]

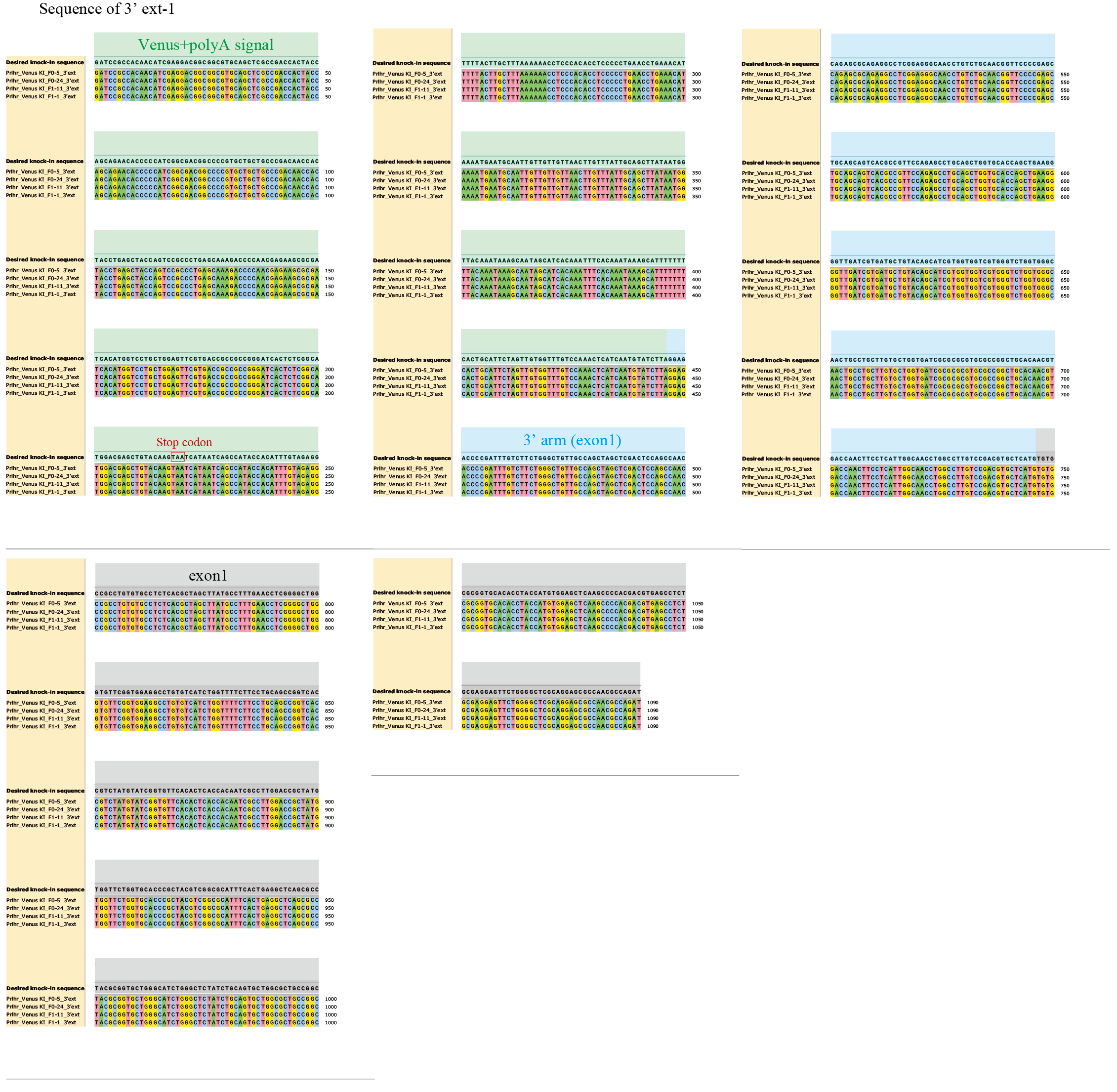

Supplement: Supplementary file 4 — Supplementary Figure S3. [file 41598_2022_24810_MOESM4_ESM.tif]

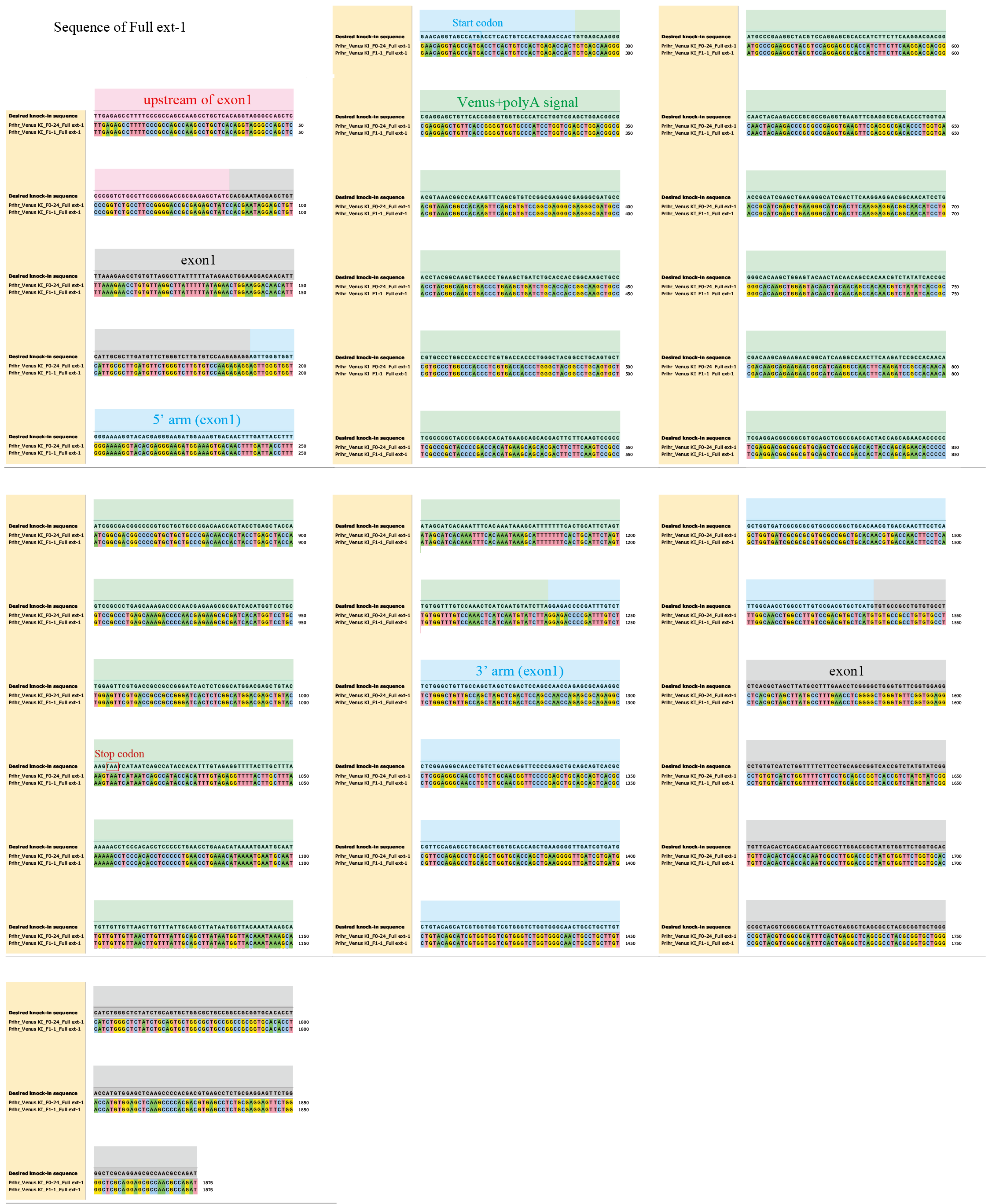

Supplement: Supplementary file 5 — Supplementary Figure S4. [file 41598_2022_24810_MOESM5_ESM.tif]

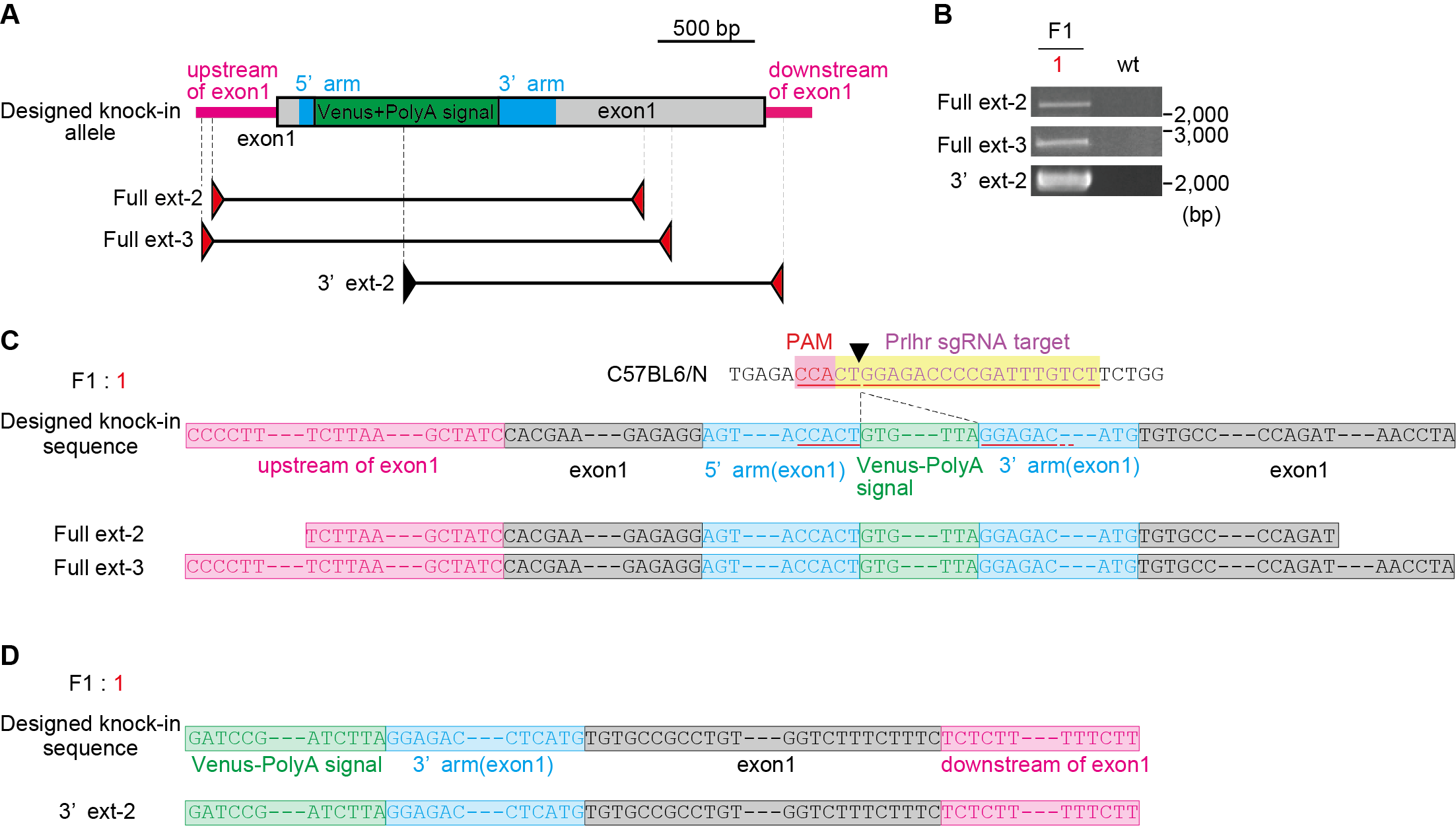

Supplement: Supplementary file 6 — Supplementary Figure S5. [file 41598_2022_24810_MOESM6_ESM.tif]

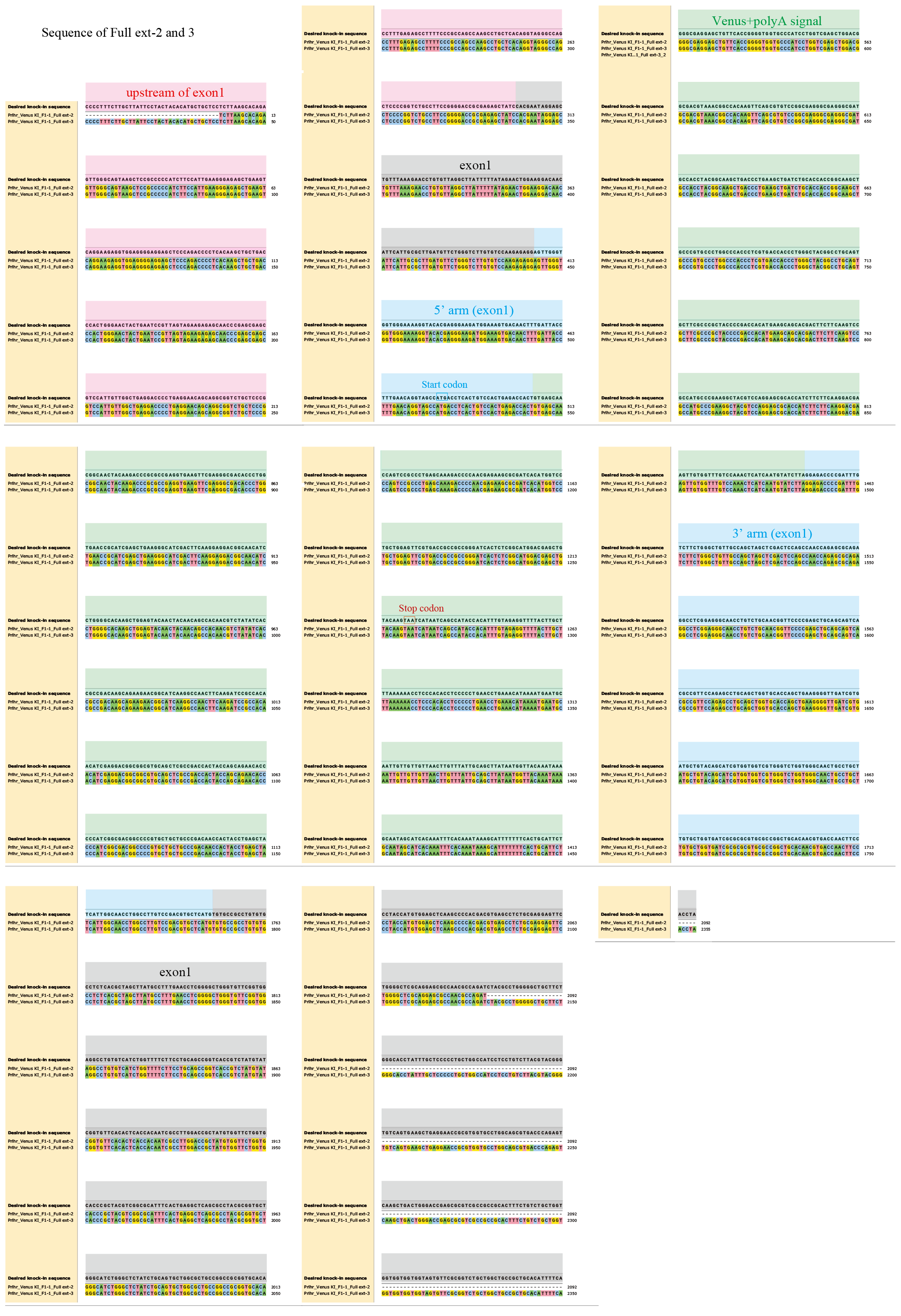

Supplement: Supplementary file 7 — Supplementary Figure S6. [file 41598_2022_24810_MOESM7_ESM.tif]

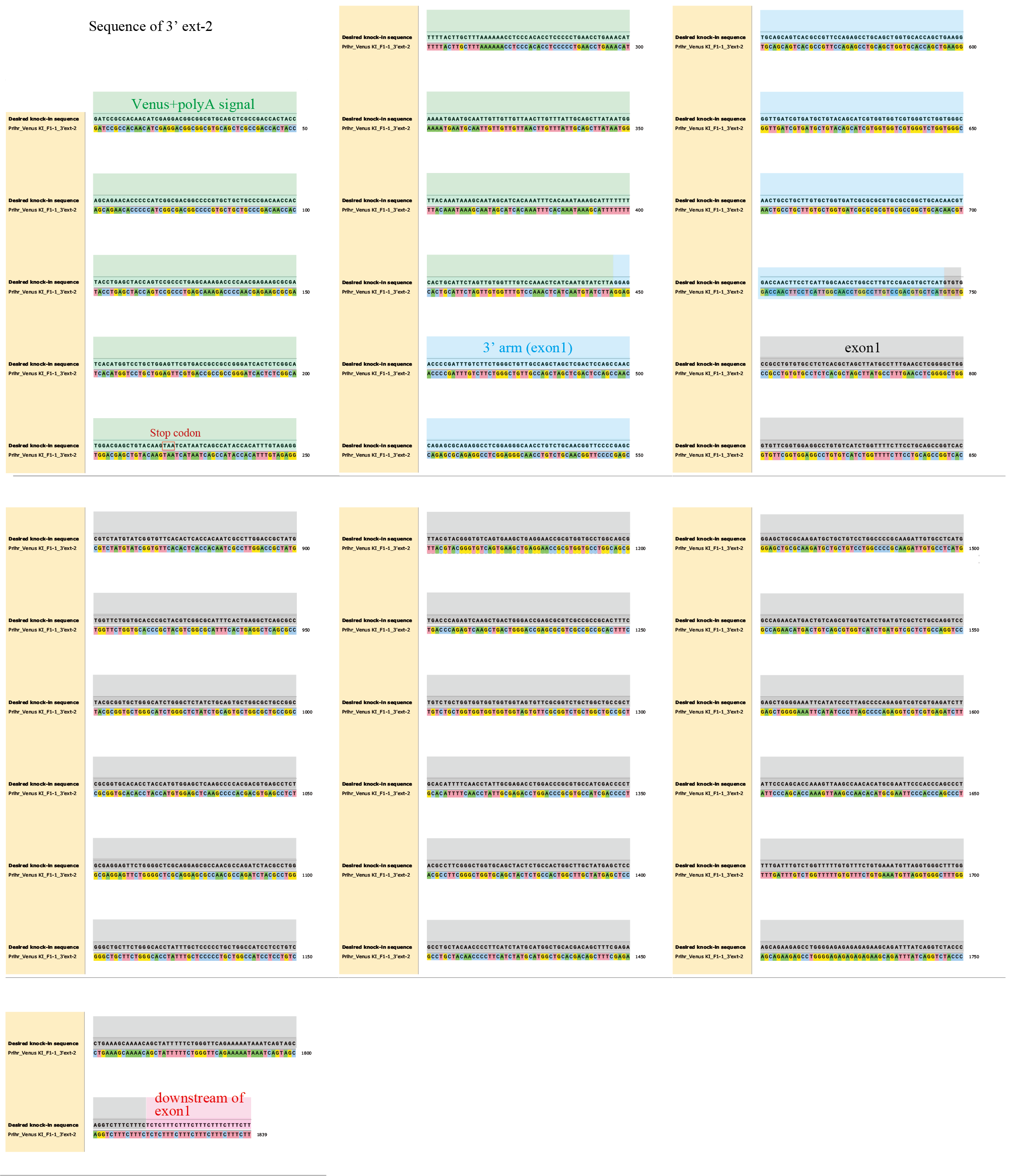

Supplement: Supplementary file 8 — Supplementary Figure S7. [file 41598_2022_24810_MOESM8_ESM.tif]

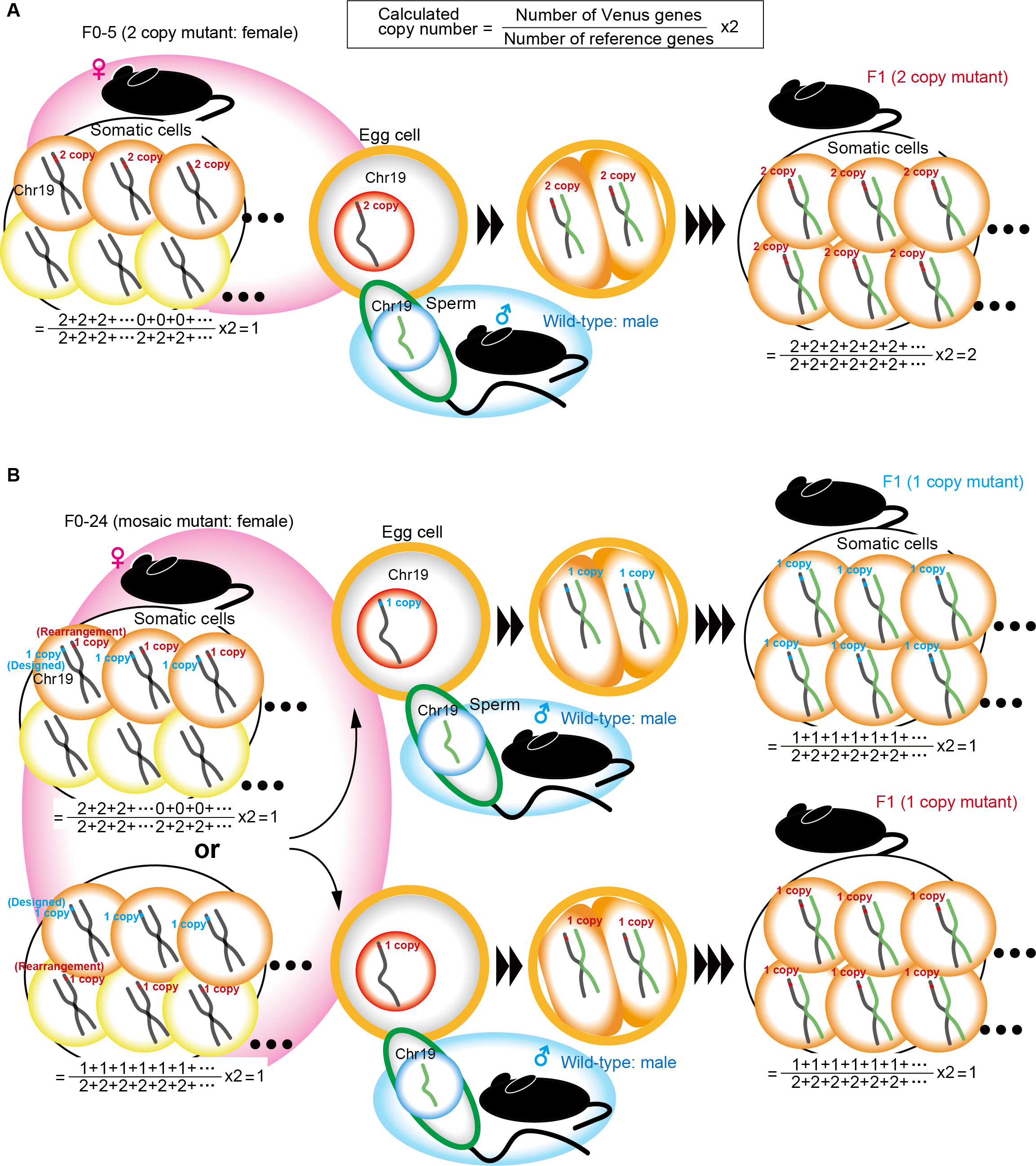

Supplement: Supplementary file 9 — Supplementary Figure S8. [file 41598_2022_24810_MOESM9_ESM.tif]

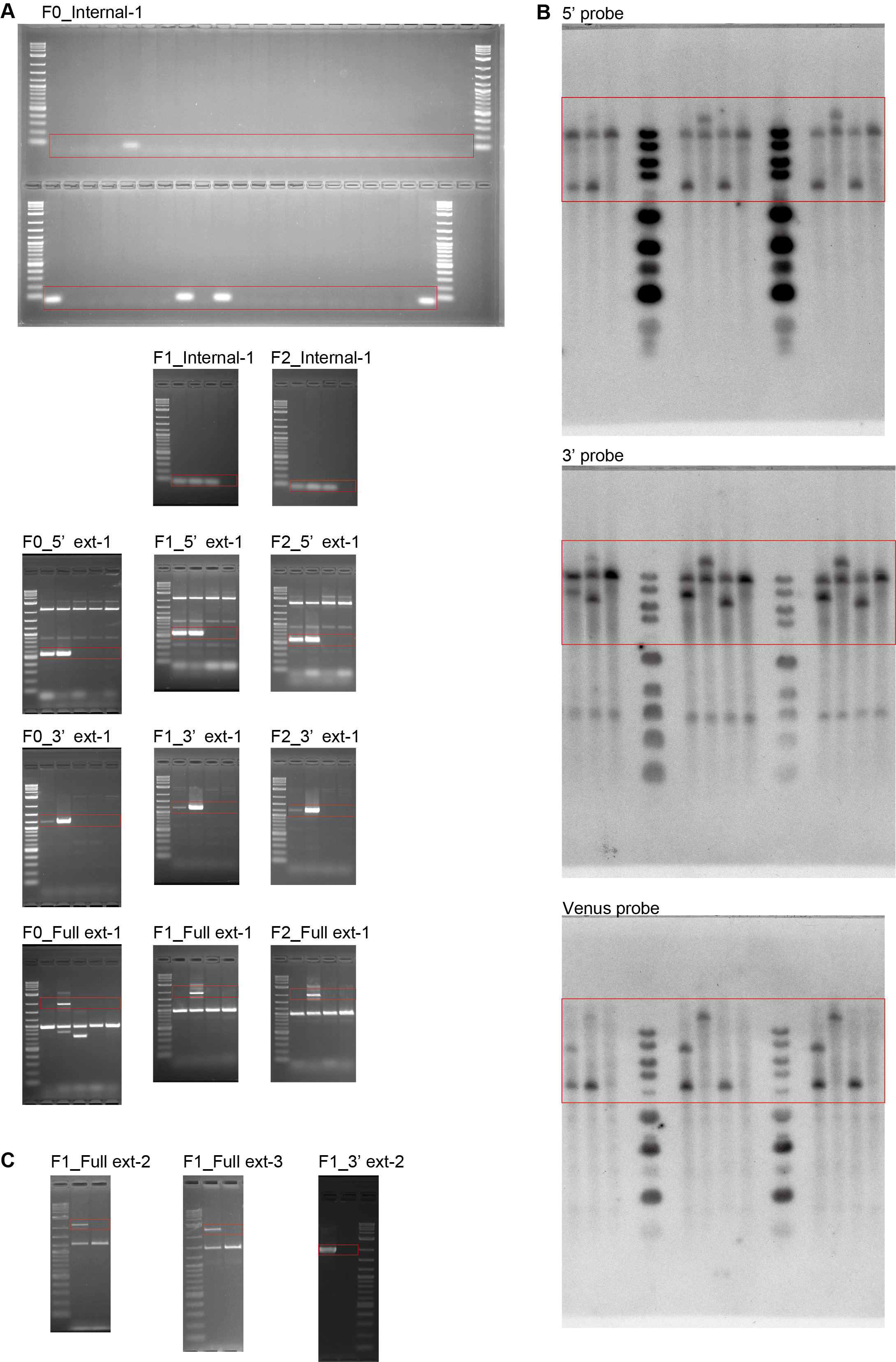

Supplement: Supplementary file 10 — Supplementary Figure S9. [file 41598_2022_24810_MOESM10_ESM.tif]

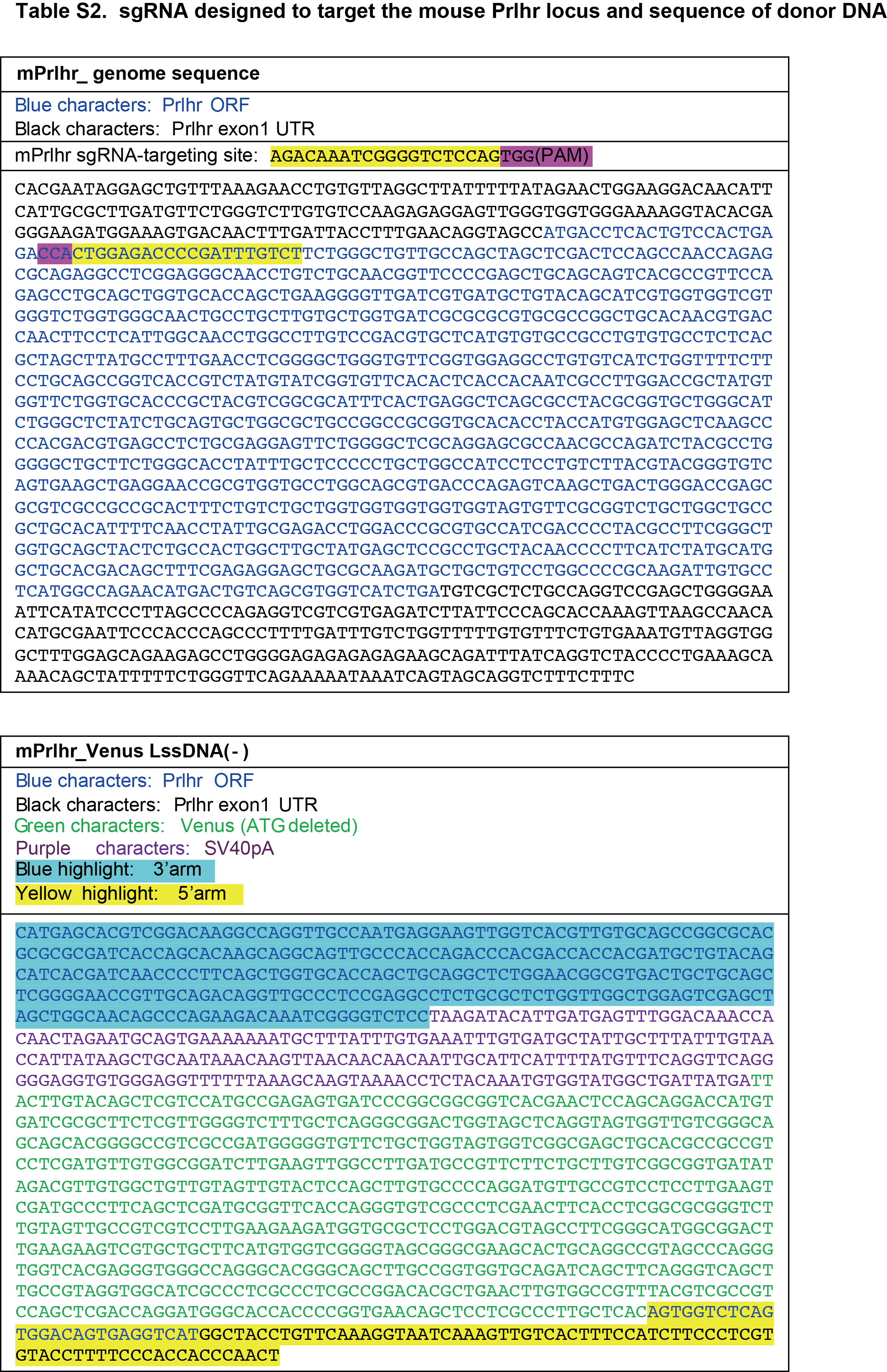

Supplement: Supplementary file 12 — Supplementary Table S2. [file 41598_2022_24810_MOESM12_ESM.tif]

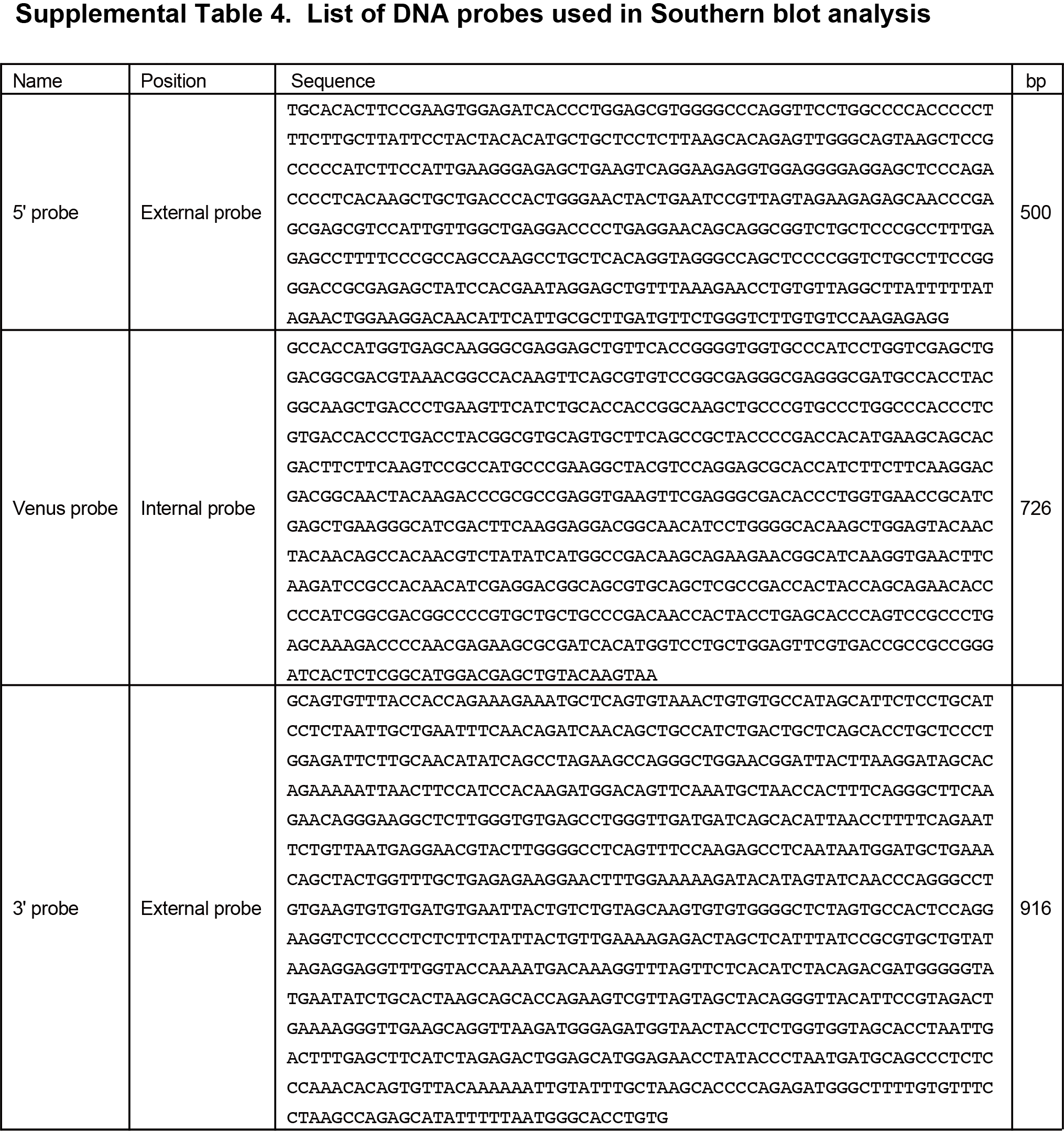

Supplement: Supplementary file 14 — Supplementary Table S4. [file 41598_2022_24810_MOESM14_ESM.tif]
